# Defensins Mediate Skin Inflammation and Itch in Psoriasis

**DOI:** 10.1016/j.jid.2026.08.014

**Published:** 2026-09-09

**Authors:** Sharan Kumar Balaji, Qin Zheng, Huasheng Yu, Joann Truong, Luchiano Giovanni Benjamin Fernando, Yining Liu, Clara Shin, Wenqin Luo, Xinzhong Dong, Xintong Dong

**Affiliations:** 1 Department of Biological Sciences, University of Texas at Dallas, Richardson, Texas, USA; 2 Department of Anesthesiology and Critical Care Medicine, Johns Hopkins University School of Medicine, Baltimore, Maryland, USA; 3 Department of Neuroscience, Perelman School of Medicine, University of Pennsylvania, Philadelphia, USA; 4 Solomon H. Snyder Department of Neuroscience, Howard Hughes Medical Institute, Johns Hopkins University School of Medicine, Baltimore, Maryland, USA

**Keywords:** Psoriasis, β-defensins, Neutrophils, Inflammation, Itch

## Abstract

Psoriasis is an inflammatory skin condition that features marked upregulation of keratinocyte-derived antimicrobial peptides, particularly β-defensins. Yet the functions of these peptides in skin inflammation remain unclear. In this study, we used a *Defensin* cluster knockout mouse to evaluate the potential roles of defensins as neuroimmune modulators in psoriasis. Deletion of the defensin gene cluster (*Def* cKO) in keratinocytes significantly attenuated imiquimod-induced psoriatic inflammation, with reduced hyperkeratosis, erythema, scaling, and expression of inflammatory cytokines and chemokines. Additionally, the *Def* cKO mice exhibited reduced neutrophil and IL-17+ TCRγδ T cell infiltration in the psoriatic skin, indicating a critical role of defensins in amplifying Th17 immunity. Behavioural analysis further revealed that loss of defensins reduced psoriatic itch, and we showed that human β-defensin 2 directly activated small-diameter Mrgpra3 neurons to elicit robust scratching. Overall, our study revealed that β-defensins play an important role in mediating inflammation and itch associated with psoriasis by activating neutrophils, type 17 immune response, and pruriceptive sensory neurons, suggesting defensin signalling as a potential therapeutic target in psoriasis.

## INTRODUCTION

Psoriasis is a chronic, immune-mediated inflammatory skin disorder that affects 2–3% of the population (Kamiya et al. 2019; Langley et al. 2005; Petit et al. 2021). It is characterized by erythema with plaques, keratinocyte (KC) hyperproliferation, neutrophil infiltration, and intense itch, all of which impact quality of life (Orsmond et al. 2021; Vijayapoopathi et al. 2023; Zhang et al. 2025). The IL-23- IL-17 pathway plays a central role in driving psoriatic inflammation (Di Cesare et al. 2009; Potestio et al. 2024). IL-23 released by dendritic cells drives the differentiation and maintenance of Th17 cells, which produce IL-17 to promote KC hyperproliferation and cytokine release, establishing a self-amplifying inflammatory loop (van der Fits et al. 2009; Hu et al. 2021; Nussbaum et al. 2021). Clinical inhibitors targeting IL-23 and IL-17 have shown considerable efficacy (Ghoreschi et al. 2021; Griffiths et al. 2021), but the immune environment is highly complex and involves other modulators that vary with disease progression. Therefore, exploring multiple signalling pathways and additional immunogenic factors is essential for a more comprehensive understanding of psoriatic inflammation.

Among the genes most strongly upregulated in psoriatic skin are members of the defensin family, a group of small, positively charged cysteine-rich antimicrobial peptides (AMPs) (Gao et al. 2021; Hollox et al. 2008; Lin et al. 2024; Morizane and Gallo 2012; Xu and Lu 2020). The first human beta-defensins, hBD2 and hBD3, were isolated from psoriatic skin (Harder et al. 2001; Harder et al. 1997), and hBD2 can serve as a biomarker for psoriasis as its levels are closely associated with the Psoriasis Area and Severity Index (PASI) score and respond quickly to anti-IL-17 treatment (Jansen et al. 2009; Kolbinger et al. 2017). Defensins are usually expressed at low levels in normal skin but are overexpressed in response to infection (Ali et al. 2001; Shelley et al. 2020; Sumikawa et al. 2006; Xu and Lu 2020), injury (Butmarc et al. 2004), and inflammation (Shelley et al. 2020). During infection, pathogen-associated molecular patterns (PAMPs) are detected by toll-like receptors (TLRs) on KCs and initiate intracellular signalling cascades that subsequently activate NF-κB, leading to the secretion of defensins and other proinflammatory mediators that aid in the clearance of pathogens (Froy 2005; Gariboldi et al. 2008; Kumar et al. 2006; Vora et al. 2004). In psoriasis, however, despite there being no invading pathogens, KCs express high levels of defensins in response to IL-17 signalling (Furue et al. 2020). In addition to their antimicrobial activity, defensins have also been shown to perform immune modulatory roles, including the recruitment and activation of mast cells, neutrophils, dendritic cells, and T cells (Biragyn et al. 2002; Dong et al. 2022; Subramanian et al. 2013). Yet despite their prevalence in psoriasis (Kolbinger et al. 2017; Tseng and Hoon 2022), whether defensins contribute to disease pathogenesis and progression remains unclear.

To elucidate the roles of defensins in psoriasis, we utilized a defensin cluster knockout mouse model (*Def* cKO) (Dong et al. 2022). The defensin genes exhibit genetic and functional redundancy, making it difficult to confirm their roles through the knockout of individual defensins (Kolar et al. 2013; Morrison et al. 2002; Navid et al. 2012; Zhou et al. 2013). The mouse defensin gene cluster is located on chromosome 8 and spans over 3 million base pairs. To delete the entire cluster, two *loxp* sites were inserted into *Defb40* and *Defb13*, which are positioned at the two ends of the cluster. The *Def^flox^* mice were bred with *Keratin14*-cre (*K14*-cre) mice to conditionally knock out all defensins in the cluster from the KCs. A prior study demonstrated that the loss of defensins resulted in dysbiosis of the skin microbiome, leading to increased *Staphylococcus* colonization and severely impaired neutrophil-mediated clearance of *Staphylococcus aureus* (Dong et al. 2022).

In this study, using the *Def* cKO mice, we show that ablation of defensins reduces psoriatic inflammation. Mice without defensins showed reduced neutrophils and IL-17-producing γδT cells. Further, we show that defensins contribute to psoriatic itch by directly activating itch sensory neurons. Collectively, these findings highlighted the neuroimmune modulatory roles of defensins in mediating psoriatic inflammation and itch.

## RESULTS

### Defensins are highly expressed by a subpopulation of inflamed keratinocytes in psoriasis patients

The increased expression of defensins in psoriasis is well documented (Harder and Schröder 2005; Jansen et al. 2009; Štrajtenberger et al. 2024). Recent whole-transcriptome analyses of patient skin samples using single-cell and spatial technologies can further clarify the spatial pattern of defensin expression in psoriatic skin, and the specific KC subpopulation that produces them. We analyzed publicly available human bulk RNA sequencing (RNAseq, GSE121212) (Tsoi et al. 2019), single cell RNAseq (scRNAseq, GSE151177) (Kim et al. 2021), and spatial transcriptomics (GSE202011) (Castillo et al. 2023) datasets to determine the location and transcriptomic features of defensin-producing KCs in psoriatic lesions. Bulk RNAseq analyses confirmed that human β-defensin genes *DEFB4A* and *DEFB103B*, which encode human β-defensin 2 (hBD2) and β-defensin 3 (hBD3), are among the most highly and significantly upregulated in psoriatic skin compared to healthy control groups (Supplementary Figure S1a) (Harder et al. 2001; Jansen et al. 2009). We then analyzed the gene expression features of defensin-producing cells using an scRNAseq dataset (Kim et al. 2021). Following the data analysis pipeline used in the original paper, we created a UMAP and categorized the cells into 12 clusters (Figure 1a, Supplementary Figure S1c). These include various immune cell types (CD4 T cells, CD8 T cells, Tregs, mature and semimature dendritic cells), melanocytes, and 4 major subclusters of KCs (Stratum basale, Stratum granulosum, Stratum spinosum, and Stratum corneum). We found that *DEFB4A* and *DEFB103B* are expressed by a subpopulation of inflamed KCs in psoriasis patients (Figure 1b). Further, we subclustered the *DEFB4A*-expressing cells from the KCs that do not express *DEFB4A* among psoriasis patients and controls. We found that *DEFB4A*-positive KCs in psoriasis also express other antimicrobial proteins, including S100 and SPRR proteins, as well as the inflammatory cytokine IL-36 (Figure 1c).

Next, we analyzed the spatial transcriptomics dataset to map the spatial distribution of these inflamed KCs in psoriatic skin. We performed dimensionality reduction and harmony-based integration analyses and annotated 9 distinct cell clusters based on marker genes used in the original study (Castillo et al. 2023). We found that *DEFB4A*-expression was enriched specifically in inflamed suprabasal KCs in psoriatic skin and was not detected in healthy skin (Figure 1d). Using violin plot and spot-level correlation analyses, we found that normalized expression of the suprabasal marker *KERATIN10 (KRT10)* and *DEFB4A* showed a partial but statistically significant positive association across tissue-associated spots (Spearman’s rho = 0.441, p = 2.21 × 10^−20^). These findings support partial spatial overlap between *KRT10*- and *DEFB4A*-expressing keratinocytes (Supplementary Figure 2a,2b). In summary, these analyses demonstrate that defensins are significantly overexpressed in the human psoriatic skin, and these defensin genes are produced by a specific subpopulation of inflamed suprabasal KCs that may play a role in the inflammation associated with psoriasis.

### Defensins mediate psoriatic inflammation

To elucidate the functions of defensins in psoriatic inflammation, we utilized a mouse model to induce psoriasis-like inflammation through the topical application of imiquimod (IMQ) cream on the nape of both *Def* cKO (Figure 2a) and wild-type (WT) mice for 7 days (van der Fits et al. 2009; Kusuba et al. 2018; Sakai et al. 2016; Wang et al. 2015). RNAseq analysis (GSE161084) (Zhang et al. 2021) confirmed that, similar to human psoriasis patients, IMQ-treated WT mouse skin showed marked elevation in the expression of *Defb4* and *Defb14*, homologs of hBD2 and hBD3 (Supplementary Figure S1b). To validate successful deletion of *Defb* genes in the *Def* cKO mouse model, we extracted RNA from WT control, 7-days IMQ-treated WT, *Def* cKO control, and 7-days IMQ-treated *Def* cKO skin and performed qPCR. Consistent with previous RNAseq results (Merleev et al. 2022; Zhang et al. 2021), IMQ-treated WT skin showed a significant increase in *Defb2*, *3*, *4*, 6 and *14* gene expression compared to WT controls, whereas *Defb1* was consistently expressed in both WT controls and IMQ-treated WT skin. In contrast, *Def* cKO control and IMQ-treated *Def* cKO mice showed almost no expression of any *Defb* genes (Figure 2b).

To determine whether murine defensins are expressed by suprabasal KCs as observed in human psoriasis, we performed RNA fluorescent in situ hybridization (RNA FISH) on IMQ-treated skin. We selected *Defb4* and *Defb14* for this study as they are homologs of *DEFB4* and *DEFB103*, which are known to be elevated in human psoriatic skin. Due to the short length of *Defb4* transcripts, we used BaseScope to chromogenically visualize *Defb4* transcripts in WT control, IMQ-treated WT, *Def* cKO control, and IMQ-treated *Def* cKO skin. IMQ-treated WT skin showed significantly elevated expression of *Defb4* (Figure 2c, blue dots) in the suprabasal layer of the epidermis compared to controls, while no transcripts were detected in control or IMQ-treated *Def* cKO skin. Similarly, *Defb14* was probed using hybridization chain reaction (HCR) RNA fluorescent in situ hybridization (RNA-FISH) in both WT control and IMQ-treated WT skin. We also found *Defb14* transcripts in the suprabasal epidermal layer in IMQ-treated WT skin compared with control (Figure 2d), and the signal was completely abolished in the *Def* cKO animals.

Next, we phenotypically compared the levels of skin inflammation in WT and *Def* cKO mice following IMQ treatment. IMQ-treated *Def* cKO mice exhibited a marked reduction in scaling compared to IMQ-treated WT mice (Figure 2e, 2h). We quantified disease severity by daily measurements of skin thickness and the cumulative psoriasis severity index (PSI), which is a combination of erythema and scaling scores. IMQ-treated *Def* cKO mice showed a significant reduction in skin thickness compared to their IMQ-treated WT counterparts starting from day 3 (p < 0.0001), and demonstrated significantly lower erythema scores from day 1 (p < 0.0001), reduced scaling from day 4 (p < 0.0067), and decreased cumulative scores from day 1 (p < 0.0008) in comparison to IMQ-treated WT animals (Figure 2f–i). We also validated these results by applying IMQ to mouse ears. Consistent with the nape skin model, IMQ-treated *Def* cKO mice showed reduced ear thickness beginning on day 4 compared with IMQ-treated WT mice (Supplementary Figure S3a, S3b). We subsequently performed histological assessment by H&E staining, further confirming a reduction in total skin thickness (epidermal and dermal) in IMQ-treated *Def* cKO animals (p = 0.0223) compared with WT (Figure 2j), supporting the conclusion that *Def* cKO mice exhibit reduced psoriatic inflammation. Overall, these findings show that defensins produced by inflamed suprabasal KCs contribute to IMQ-driven psoriatic inflammation.

### IMQ treatment alters the skin microbiome in WT and *Def* cKO mice

Given the antimicrobial function of defensins, we examined whether changes in the skin microbiome could contribute to the reduced IMQ-induced inflammation observed in *Def* cKO mice. To address this, we analyzed the skin microbiome of WT and *Def* cKO mice under control and IMQ-treated conditions. Mice were weaned and housed with littermates of the same genotype for 7 weeks before IMQ treatment. Skin swabs were then collected from the nape skin and analyzed by 16S rRNA sequencing (Bolyen et al. 2019; Fyhrquist et al. 2019; Grogan et al. 2019). Beta diversity analysis showed that WT and *Def* cKO control samples clustered closely together, whereas IMQ-treated WT and *Def* cKO samples had distinct microbiomes (Supplementary Figure S4a). Alpha diversity, measured by Shannon indices, was significantly reduced after IMQ treatment in both WT mice and *Def* cKO mice, indicating that IMQ-induced inflammation is associated with a loss of microbial diversity. However, Shannon diversity was not significantly different between IMQ-treated WT and *Def* cKO samples (Supplementary Figure S4b). Taxonomic analysis showed that IMQ-treated WT mice had an increased relative abundance of *Staphylococcus*, whereas IMQ-treated *Def* cKO mice showed increased relative abundance of *Staphylococcus*, *Streptococcus,* and *Corynebacterium* (Supplementary Figure S4c). Together, these findings indicate that IMQ treatment induces marked changes in the skin microbiome in both WT and *Def* cKO mice, but the reduction in microbial alpha diversity occurs in both WT and *Def* cKO mice and therefore does not alone explain the reduced inflammatory phenotype observed in *Def* cKO animals.

### Defensins mediate neutrophil infiltration in psoriasis

To understand how defensin loss-of-function alters the immune environment in psoriatic skin, we examined the expression of key proinflammatory mediators associated with psoriasis. qPCR analysis revealed that IMQ-treated *Def* cKO skin exhibited a marked reduction in the expression of neutrophil-associated genes, including *Il1b*, *Cxcl2*, *Tnf*, *S100a8*, and *S100a9*, compared with IMQ-treated WT skin (Figure 3a) (Man et al. 2023; Sato et al. 2020; Sieminska et al. 2024). These findings suggest that defensins may contribute to psoriasis by promoting neutrophilic inflammation.

The accumulation of neutrophils in microabscesses within the epidermis is a key feature of psoriasis (Chiang et al. 2019). We next investigated whether defensins influence neutrophil recruitment in IMQ-treated skin after day 7 using flow cytometry. Neutrophils were gated as CD45^+^CD11b^+^Ly6g^+^ cells (Figure 3b). IMQ-treated *Def* cKO skin showed a significant decrease in the percentage of neutrophils compared to IMQ-treated WT mice (p < 0.0001) (Figure 3b), which correlates with lower expression of neutrophil-specific genes (Figure 3a). To further validate these findings histologically, we performed immunofluorescence staining using an anti-Ly6g antibody on WT control, IMQ-treated WT, *Def* cKO control, and IMQ-treated *Def* cKO skin. We quantified the number of Ly6g+ cells in both the epidermis and the full skin. Consistent with flow cytometry results, IMQ-treated *Def* cKO skin showed a significant decrease in the overall count of Ly6g+ cells compared to IMQ-treated WT skin (Figure 3c). Furthermore, Ly6g+ positive cells in the epidermis were also reduced in IMQ-treated *Def* cKO mice compared to WT. Taken together, these results demonstrate that defensins are crucial mediators of neutrophil recruitment and accumulation in psoriatic skin. The loss of defensins in *Def* cKO mice impairs neutrophil infiltration, reduces inflammation, and reduces epidermal microabscess formation, highlighting the crucial role of defensins in driving neutrophilic inflammation in psoriasis.

### Defensins induce IL-17 secretion from neutrophils and mediate IL-17+ TCRγδ T cell infiltration in psoriasis

IL-17 and IL-23 are essential cytokines involved in the progression of psoriasis. Our qPCR results showed that IMQ-treated *Def* cKO animals exhibited significant downregulation of *Il17a*, *Il17f*, and *Il23* compared with IMQ-treated WT animals (Figure 3a). Previous studies have shown that neutrophils can serve as a source of IL-17 in psoriatic lesions and other inflammatory conditions (Dyring-Andersen et al. 2017; Li et al. 2010; Lin et al. 2011). To investigate whether defensins can stimulate IL-17 release from neutrophils, we cultured bone-marrow-derived neutrophils from WT mice and stimulated them with recombinant human β-defensin 2 and 3 (hBD2 and hBD3, 10 μM) for 12 hours (McGill et al. 2021). We then collected the supernatants and performed an enzyme-linked immunosorbent assay (ELISA). Our findings revealed that cultured bone marrow neutrophils showed a significant increase in IL-17 secretion upon stimulation with hBD3 and lipopolysaccharide (LPS), which was used as a positive control (Ferretti et al. 2003; Hu et al. 2017) (Figure 3d). Because neutrophils are short-lived and susceptible to cell death during prolonged culture, we wanted to determine whether IL-17 release is due to neutrophil activation upon hBD3 stimulation or due to cell death. To assess cytotoxicity, bone marrow–derived neutrophils were stimulated with hBD2, hBD3, LPS, or phorbol 12-myristate 13-acetate (PMA). PMA was included as a positive control because it induces NETosis (Inozemtsev et al. 2023). LDH analysis showed that 12-hour treatment with LPS, hBD2, or hBD3 increased cytotoxicity compared with unstimulated controls; however, the magnitude of LDH release was substantially lower than that induced by PMA (Supplementary Figure S5) and stimulation with PMA does not show any significant increase in IL-17 levels (Figure 3d), suggesting that the IL-17 detected by ELISA following hBD3 treatment was not primarily due to cell death and instead is consistent with hBD3-mediated neutrophil activation.

To evaluate the effects of defensin loss-of-function on T cell populations, we performed flow cytometry analyses to quantify total T cells (CD3+), CD4+, CD8+, and TCRγδ T cell populations. We were particularly interested in IL-17+ TCRγδ T cells, since this population had been known to be expanded in psoriasis by responding to IL-23, producing IL-17, and driving inflammation (Cai et al. 2011; Qi et al. 2021). We found that IMQ-treated *Def* cKO animals exhibited a significant decrease in the percentage of IL-17+ TCRγδ T cells compared to IMQ-treated WT animals (Figure 3e), while no changes were observed in the other T cell populations (Supplementary Figure S6a–d). These results lead to an overall model where defensins promote IL-17 production by neutrophils and facilitate the infiltration of IL-17+ TCRγδ T cells, thereby amplifying the IL-17 signalling pathway and exacerbating inflammation in psoriasis.

### Defensins activate Mrgpra3 neurons to mediate psoriatic itch

Itch is a common symptom among individuals with psoriasis and tends to worsen as the disease progresses (Elewski et al. 2019; Komiya et al. 2020; Yosipovitch et al. 2018). Interestingly, defensins have been shown to activate members of the Mas-related G-protein-coupled receptor (Mrgpr) family—particularly Mrgpra3 and Mrgprc11—which are expressed in sensory neurons responsible for itch (Bader et al. 2014; Liu et al. 2009; McNeil and Dong 2014; Meixiong et al. 2019; Tseng and Hoon 2022; Zhang and McNeil 2019a). Therefore, we sought to determine whether defensins mediate itch associated with psoriasis. We treated the nape skin of both WT and *Def* cKO mice with IMQ for 7 days and recorded the animals’ scratching behaviour. Itch behaviour was quantified by both manual counting of scratch bouts over a 30-minute period and an automated machine-learning algorithm, Scratch-AID (Yu et al. 2022). Notably, we observed that *Def* cKO mice treated with IMQ exhibited less scratching behaviour compared to the WT mice (Figure 4a and 4b).

We next investigated whether Mrgpra3+ neurons are crucial for defensin-mediated psoriatic itch using a *Mrgpra3*^DTA^ mouse model in which all Mrgpra3+ neurons are selectively ablated via Cre-dependent expression of diphtheria toxin subunit A (DTA). *Mrgpra3*^DTA^ mice subjected to IMQ showed a marked decrease in itch behaviour compared with WT mice (Figure 4c). ScratchAID-based quantification further showed that IMQ-treated *Mrgpra3*^DTA^ mice exhibited significantly reduced total scratch time compared with WT mice, consistent with the manual scratch bouts (Figure 4d). Next, we directly assessed the ability of defensins to cause itch by administering mBD4, hBD2 and hBD3 peptides intradermally into the cheeks of WT and *Mrgpra3*^DTA^ mice. We used human defensins hBD2 and hBD3 because they are functional homologs of mBD4 and mBD14, and prior studies have shown that human β-defensins can activate mouse Mrgpr receptors, which are homologous to human MRGPRs, with similar EC50 values (Dong et al. 2022; Tseng and Hoon 2022; Zhang et al. 2026). Consistent with previous reports (Tseng and Hoon 2022; Zhang and McNeil 2019), mBD4, hBD2 and hBD3 elicited scratching responses in the WT mice, while the ablation of Mrgpra3+ neurons significantly reduced both the scratch bouts and the total scratch time to defensin injections, supporting our hypothesis that defensins trigger itch by activating Mrgpra3+ neurons (Figure 4e–j). To further assess whether defensins activate sensory neurons directly to provoke itch or do so indirectly through immune cell activation, we isolated dorsal root ganglia (DRG) from *Pirt*^Salsa^ mice, in which all DRG neurons express a Tdtomato marker and a genetically encoded calcium indicator GCaMP6f, and stimulated the neurons with hBD2. This *in vitro* calcium imaging experiment demonstrated that hBD2 activated small-diameter neurons (Figure 5a). Among the hBD2-activated neurons, 41.97 ± 15.99 % neurons also responded to the Mrgpra3 agonist chloroquine (CQ) (Figure 5a and 5b). We also examined the overlap between hBD2-responsive neurons with the NP1/MrgprD+ neurons, which are activated by β-alanine and have been implicated in itch and pain (Guo et al. 2023; Liu et al. 2012), as well as NP3/serotonin-responsive neurons, which are also involved in itch signalling (Cevikbas and Lerner 2020). In vitro calcium imaging showed that smaller portions of hBD2-responsive neurons also responded to β-alanine (16.16 ± 8.66%) or serotonin (19.05 ± 17.98%) (Supplementary Figure S7a–d). Together, these three itch neuron populations account for roughly 77% of all hBD2-responsive neurons, indicating that itch is the primary sensory modality activated by hBD2. Similarly, we also isolated DRG from WT and *Mrgpra3*^DTA^ mice and performed calcium imaging with Fluo-4, a dye that measures intracellular calcium. We found that the total number of hBD2-activated neurons was reduced in the *Mrgpra3*^DTA^ mice as compared to WT (Figure 5c, Supplementary Figure S8a), supporting the notion that hBD2 directly activates Mrgpra3+ neurons. We further assessed the ability of defensins to activate sensory neurons using *in vivo* calcium imaging by injecting hBD2 and CQ into the hind paw of *Pirt*^GCaMP6s^ mice, which express the genetically encoded Ca^2+^ indicator GCaMP6s in all DRG neurons. Using *in vivo* calcium imaging of the lumbar 4 (L4) DRG, we observed that hBD2 elicited robust neuronal activation. Notably, 62.89 ± 15.98% of hBD2-responsive neurons also responded to CQ, indicating substantial overlap with the Mrgpra3-positive pruriceptive population and supporting our *in vitro* findings (Figure 5d). Together, these results show that hBD2 can elicit itch by directly activating the Mrgpra3+ NP2 itch neurons. These results are consistent with the proposed role of defensins as itch mediators (Tseng and Hoon 2022).

Next, we investigated the localization of defensin-expressing KCs with respect to Mrgpra3+ neurons innervating the skin. We performed HCR RNA-FISH for *Defb14* on IMQ-treated *Mrgpra3*^Tdtomato^ mice, in which Mrgpra3+ nerve terminals in the skin are visualized with Tdtomato. We found that *Defb14* transcripts are localized near Mrgpra3+ neurons, confirming that these peptides can potentially interact directly with itch sensory neurons (Supplementary Figure S8b). Previous research showed that activated Mrgpra3+ neurons could secrete neuropeptides and regulate skin immunity (Inclan-Rico et al. 2024). To determine whether Mrgpra3^+^ neurons are necessary for psoriatic inflammation, we investigated IMQ-treated *Mrgpra3*^DTA^ mice, in which Mrgpra3^+^ neurons are selectively ablated. These mice exhibited a mild but statistically significant reduction in skin thickness compared with IMQ-treated WT controls, but their PSI scores were not significantly different (Supplementary Figure S9a–e), indicating a more specialized role for Mrgpra3^+^ neurons in mediating psoriatic itch rather than driving inflammation. Together, these results suggest that defensins directly activate Mrgpra3+ itch sensory neurons to induce psoriatic itch.

## DISCUSSION

In this study, we demonstrated that defensins secreted by inflamed KCs act as critical activators of immune cells and sensory neurons, driving psoriatic inflammation and itch. The immune-modulatory roles of defensins have been recognized for decades (Harder et al. 1997; Morizane and Gallo 2012; Semple and Dorin 2012; Yang et al. 1999). But the functional significance of their increased expression in psoriasis has long remained unresolved due to significant genetic redundancy within this locus (Morrison et al. 2002; Navid et al. 2012). By deleting the entire defensin cluster in KCs, we directly examined how the lack of these AMPs affects psoriatic inflammation and itch *in vivo*.

Our results showed that defensins are critical for the development of IMQ-induced psoriasis. *Def* cKO animals showed a significant reduction in epidermal hyperkeratosis, lower expression of inflammatory mediators, and reduced immune cell infiltration. Through transcriptomic analysis, we found that defensin expression is restricted to a distinct population of inflamed suprabasal KCs that co-expresses AMPs, including S100 proteins, small proline-rich region (SPRR) proteins, and IL-36 family cytokines. These inflammatory molecules are well established in the context of psoriatic inflammation and barrier stress (Foster et al. 2014; Hänel et al. 2013; Saito-Sasaki and Sawada 2023; Tian et al. 2021). These AMP-expressing KCs within this spatially localized inflammatory niche are potentially important drivers of psoriatic pathology.

A previous study identified neutrophils as key effector cells of defensin (Dong et al. 2022). Neutrophils are abundant innate immune cells in circulation and perform a wide range of immune functions (Fox et al. 2010; Hermosilla et al. 2014; Kienle and Lämmermann 2016; Radic and Marion 2013). Neutrophils contribute to psoriatic inflammation by releasing cytokines, activating other immune cells, and forming neutrophil extracellular traps (NETs), and their accumulation in Munro’s microabscesses is a hallmark of the disease (Chiang et al. 2019; Czerwińska and Owczarczyk-Saczonek 2022; Wang and Jin 2020; Wang and Shi 2023). We also found that loss of defensins reduced neutrophil infiltration in the skin, revealing defensins as key epithelial signals that mediate neutrophil accumulation in psoriatic lesions. Neutrophils also express IL-17 in inflammatory conditions (Dyring-Andersen et al. 2017; Li et al. 2010; Lin et al. 2011), and the IL-23/IL-17 axis is essential for the progression of psoriasis. Th17 cells are the major producers of IL-17a and IL-17f, which drive KC proliferation and promote the expression of cytokines and AMPs (Blauvelt and Chiricozzi 2018; Martin et al. 2013; Mosca et al. 2021), creating a self-amplifying loop of inflammation. We found that defensins directly induce IL-17 production by neutrophils, and that the percentages of IL-17+ TCRγδ T cells were lower in the skin of *Def* cKO animals following IMQ treatment. These results suggest that defensins affect both the innate and adaptive arms of the IL-23/IL-17 pathway, further emphasising their importance as regulators of psoriatic inflammation.

In addition to modulating immune responses, defensins directly activate sensory neurons to drive psoriatic itch. Itch in psoriasis is among the most disruptive symptoms reported by psoriasis patients, yet its mechanistic basis remains incompletely understood (Komiya et al. 2020; Taliercio et al. 2021). Previous studies have shown that β-defensins can activate sensory neurons via Mrgprc11 and Mrgpra3 in mice (Bader et al. 2014; Meixiong and Dong 2017), but a direct role of defensins in psoriatic itch has not been demonstrated. Here, we show that loss of defensins substantially reduced scratching behaviour and that genetic ablation of Mrgpra3 neurons mimicked this effect.

Although *Def* cKO mice showed significantly reduced scratching after IMQ treatment, scratching was not completely abolished. This residual itch likely reflects the complex inflammatory environment of psoriatic skin. Previous studies have identified several itch mediators in psoriasis, including neuropeptides such as substance P, nerve growth factor (NGF), which can increase cutaneous nerve density and aggravate histamine-independent itch, and cytokines such as IL-31, TSLP, and IL-2. These mediators are elevated in lesional psoriatic skin and may contribute to itch independently of defensins (Komiya et al. 2020; Reich and Szepietowski 2007). Thus, defensin-independent pruritogenic pathways likely remain active in *Def* cKO mice and contribute to the residual scratching observed after IMQ treatment. In contrast, hBD2-evoked scratching was strongly reduced in *Mrgpra3*^DTA^ mice, indicating that Mrgpra3+ neurons are the major neuronal pathway mediating acute defensin-induced itch.

Both *in vivo* and *in vitro* calcium imaging confirmed that human β-defensins activated small-diameter sensory neurons, many of which are Mrgpra3-responsive. These results align with earlier studies showing that Mrgpra3+ sensory neurons are the primary pruriceptors in a variety of inflammatory skin conditions (Fujii et al. 2024; Inclan-Rico et al. 2024; Liu et al. 2025). In addition to Mrgpra3+ NP2 neurons, hBD2 also activated a subset of β-alanine- or serotonin-responsive populations (NP1 and NP3 neurons), suggesting that hBD2-responsive neurons are heterogeneous.

In addition to direct neuronal activation, we also explored the possibility that defensins cause psoriatic inflammation and itch through mast cell activation. Previous studies have shown that β-defensins can trigger mast cell degranulation through the Mrgprb2 receptor (Liu et al. 2025; Shao et al. 2022; Subramanian et al. 2013; Tseng and Hoon 2022). We used the IMQ model on the nape skin of WT and *Mrgprb2* KO mice and monitored erythema, scaling, cumulative PSI, and skin thickness over time. IMQ-treated *Mrgprb2* KO mutants did not show significant phenotype compared to IMQ-treated WT (Supplementary Figure S10a–e). These data suggest that mast cells might not be the primary defensin effector cells, and that other defensin signalling pathways might be involved in mediating psoriatic inflammation.

Our findings position defensins at the intersection of KC inflammation, innate immune activation, and sensory neuron excitability. However, several open questions remain. First, we are currently investigating the mechanisms by which defensins regulate neutrophil activities in psoriasis. Recent high-throughput sequencing revealed various circulating neutrophil states and tissue-specificities (Hatje et al. 2025; Nishide et al. 2025; Xie et al. 2020). Transcriptomics analysis of neutrophils isolated from IMQ-treated skin of WT and *Def* cKO mice is required to further understand the effects of defensins on neutrophil signalling and gene expression. Second, while our study solved the genetic redundancy problem using a cluster KO approach, we could not resolve the effects of individual defensins. In addition to *Defb4* and *Defb14*, other defensins and AMP families may cooperate to sustain chronic inflammation and itch. Third, although our 16S analysis showed that IMQ treatment altered the skin microbiome in both WT and *Def* cKO mice, these data do not establish whether microbial changes contribute to the reduced inflammation or itch observed in *Def* cKO animals. Future studies using antibiotic treatment, gnotobiotic models, and microbial reconstitution will be needed to determine whether altered microbial communities contribute to inflammation and itch in psoriasis. Fourth, psoriasis involves a wide array of immune populations, including dendritic cells, γδ T cells, innate lymphoid cells, and macrophages (Kamata and Tada 2022; Nestle et al. 2009). In our study, we showed that *Def* cKO animals showed reduced expression of IL-23, which is primarily released by dendritic cells, and it will be important to determine how defensin signalling intersects with these cellular networks. Fifth, a recent study found that Mrgpra3+ neurons regulate the expansion of IL-17+ TCRγδ T cells during helminth infections (Inclan-Rico et al. 2024), and whether these neurons play a comparable role in psoriasis remains to be investigated. Finally, our findings also have implications beyond psoriasis. Dysregulated defensin expression and neutrophil activation are features of several chronic inflammatory skin conditions, including hidradenitis suppurativa (Giamarellos-Bourboulis et al. 2016) and atopic dermatitis (Chieosilapatham et al. 2017). The identification of defensins as inflammatory mediators and pruritogens suggests that modifying defensin–neuroimmune interactions could represent a therapeutic strategy for chronic itch disorders.

In conclusion, our findings establish defensins as important neuroimmune modulators in psoriasis. By activating neutrophils, promoting the IL-17 signalling axis, and directly activating pruriceptors, KC-derived defensins serve as key regulators of both inflammation and itch. These findings illuminate, to our knowledge, a previously unreported inflammatory pathway mediated by defensins and point toward new therapeutic opportunities targeting defensin-mediated pathways in psoriasis.

## MATERIAL AND METHODS

### Mouse lines and IMQ-induced psoriasis model

The mouse models used in this study are C57BL/6J, *Def* cKO, *Mrgpra3*^DTA^, *Mrgpra3*^Tdt^, *Pirt*^Tdt^, *Pirt*^Gcamp6s^, *Pirt*^Salsa,^ and *Nav1.8*^Tdt^. *Def* cKO mouse was created by Dr. Xintong Dong (Dong et al. 2022). C57BL/6J, *ROSA*^Tdt^, *ROSA*^DTA^, *ROSA*^Salsa^, and *Nav1.8*-cre were obtained from The Jackson Laboratory. *Pirt*-cre, *ROSA*^Gcamp6s^ and *Mrgpra3*-cre lines were obtained from Johns Hopkins University (Han et al. 2013; Kim et al. 2008; Zhang et al. 2024). Experiments were performed with IACUC-approved protocols. Age- and sex-matched mice were generally treated between 7 and 10 weeks of age and were used in all experiments.

To induce psoriasis, age-matched (7-10 weeks old) mice were anesthetized with isoflurane, their nape skin was shaved on day 0, and 5% imiquimod (IMQ) (Padagis) was applied topically to the nape and ear for 7 days. The phenotype was observed by taking images and measuring the skin thickness on each day. The images were scored by a blinded experimenter on a scale of 1 to 5 to calculate the psoriasis severity index (PSI) for erythema and scaling. The mice were euthanized on day 8, and the skin was extracted using an 8mm biopsy punch for downstream experiments.

### Behaviour studies

In the nape model, WT C57BL/6J, *Def* cKO, *Mrgpra3*^DTA^ (7-10 weeks old) were topically treated with 5% IMQ for 7 days. The mice were habituated to a scratchAID (Automatic itch detection) chamber for 3 days (15 minutes each), and scratching behaviours were recorded for 30 minutes on day 0 and day 8 in the morning. The behavioural setup consisted of a laptop connected to a Logitech webcam, and recordings were acquired using Logitech Capture software. For the cheek injections, WT and *Mrgpra3*^DTA^ mutants (7-10 weeks old) were habituated in a scratchAID chamber for 3 days (15 minutes per day). 20 μL of mBD4, hBD-2 and hBD-3 (100 μM in PBS) or saline are directly injected into the cheeks of WT and *Mrgpra3*^DTA^ mutants, and the scratch bouts were recorded over 30 minutes. A scratching behaviour was defined as a single hind paw stroking the site of injection. The total scratch bouts were counted manually, and the total scratch time was evaluated using deep learning software (Yu et al. 2022).

### Flow Cytometry

8mm skin biopsies were cut into small pieces and digested in 4.5 ml RPMI without fetal bovine serum, 500 μL Liberase TL (1.67 Wunsch units/mL) (Roche), and 10% DNase 1 (Sigma-Aldrich) and incubated for 1 hour and 30 minutes at 37 °C on a rotor. The samples were then passed through a 100-micron filter to generate a cell suspension, which was then centrifuged at 300g for 5 minutes. The cell suspension was washed with PBS and stained using Live/Dead Fixable Aqua Dead Cell Stain Kit (Thermo Fisher) for 30 minutes. The cells were centrifuged and resuspended in FACS buffer (2% FBS in PBS). Cells were then treated with Monocyte blocker (BioLegend) and Fc block (BioLegend) for 5 mins before adding the antibody cocktail: CD45-PerCP/Cyanine 5.5 (QA17A26, BioLegend), CD11b-PE-eFluor 610 (M1/70, Invitrogen), Ly6G-Brilliant Violet 421 (1A8, BioLegend), Siglec-F-FITC (S17007L, BioLegend), CD3-FITC (17A2, BioLegend), CD4-Brilliant Violet 605 (RM4-4, BioLegend), CD8b-APC (YTS156.7.7, BioLegend), TCR γ/δ-Brilliant Violet 421 (GL3, BioLegend) and IL-17F-PE (9D3.1C8, BioLegend) and incubated for 30 minutes in dark at 4 °C. Cells were then passed through a 40-micron filter and data were collected using BD-LSR Fortessa and analysed using FlowJo (BD). Neutrophils were gated as live CD45^+^CD11b^+^Ly6G^+,^ and IL-17 T cells were gated as live CD45^+^CD3^+^TCR γ/δ^+^IL-17^+^.

### Histology

Skin samples were fixed in 4% paraformaldehyde (PFA) for 2 days, then in 70% ethanol overnight, embedded in paraffin, and sectioned at 10 micron. The sections were then deparaffinized and stained with haematoxylin and eosin (H&E) according to the manufacturer’s protocol (Vector Laboratories). The slides were imaged using a Keyence BZ-X800 microscope (Keyence). For immunofluorescence, skin samples were fixed in 4% paraformaldehyde (PFA) for 2 days, followed by 20% sucrose for 2 days and then 30% sucrose for 2 days. Then, tissues are embedded in OCT and sectioned at 10 micron. The slides with the tissues are baked at 65°C for 1 hour and blocked in a blocking buffer (5% goat serum, 1%BSA, 0.2% Triton in PBS) for 1 hour. The sections were then stained with a 1:200 dilution of rabbit anti-mouse Ly6G (E6Z1T, Cell Signaling Technology) in antibody blocking buffer (5% goat serum, 1% BSA) overnight at 4 °C. The next day, sections were incubated for 1 hour at room temperature with a 1:300 dilution of Alexa Fluor 488 goat anti-rabbit IgG (Thermo Fisher Scientific, for neutrophil staining), then rinsed 3 times with PBS. The slides were dried and mounted with Fluoromount-G with DAPI (Invitrogen). The slides were imaged using a ZEISS Axio Imager Z2 confocal microscope, and the images were analysed using ImageJ (Fiji) (Schindelin et al. 2012).

### HCR RNA-fluorescence *in situ* hybridisation & BaseScope

10 micron cryosectioned skin tissues were fixed by immersing slides in ice-cold 4% paraformaldehyde (PFA) for 15 minutes at 4 °C, followed by a series of ethanol washes of different percentages (50%, 70% and 100%). The sections were probed for *Defb14* and hybridized overnight using a Boekel Scientific RapidFISH Slide Hybridizer. Further amplification and labelling were performed according to the manufacturer’s instructions (Molecular Instruments). The images were taken using a ZEISS Axio Imager Z2 confocal microscope. For BaseScope, 10 μM paraffin-embedded skin tissues were deparaffinized and probed for *Defb4* and *Krt16*, and amplified according to the manufacturer’s instructions (ACD Bio). The slides were imaged using a Keyence BZ-X800 microscope (Keyence).

### RNA extraction and RT-PCR

Skin tissues were homogenized in TRIzol reagent. Then chloroform was added in a 1:5 ratio, mixed, and centrifuged for 15 mins at 21000g at 4°C. The top layer containing RNA was transferred to a new tube, and an equal volume of 100% ethanol was added. Then, the entire solution was transferred to a Qiagen spin column, and RNA was extracted using the Qiagen RNeasy Mini Kit (Qiagen) according to the manufacturer’s instructions. The RNA was quantified using a Nanodrop, and 1000 ng of RNA was used as the template to generate cDNA with the High-Capacity cDNA Reverse Transcription kit (Applied Biosystems). Quantitative PCR (qPCR) was performed using TaqMan Fast Advanced Master Mix (Applied Biosystems) and TaqMan probes for the following genes (*Defb1*, *Defb2*, *Defb3*, *Defb4*, *Defb6, Defb14*, *Il17a*, *Il17f*, *Il11b*, *Il23*, *Cxcl2*, *S100a8*, *S100a9, Tnf*, and *Actb*) and performed using a Quant Studio 3 RT-PCR system (Applied Biosystems). Gene expression was normalized to *Actb* using the ΔCt method.

### 16S Sequencing

Skin microbiome samples were collected by swabbing the nape skin of WT and *Def* cKO mice under control and IMQ-treated conditions. Microbial DNA was extracted from the swabs using bead beating followed by phenol-chloroform extraction. Briefly, each swab was transferred to a sterile bead-beating tube containing 0.3 g glass beads, extraction buffer containing 200 mM NaCl, 200 mM Tris, and 20 mM EDTA, 20% sodium dodecyl sulfate, and phenol:chloroform:isoamyl alcohol. Swabs were pressed against the tube wall to release sample material, chilled on ice, and homogenized by bead beating for 2 minutes at 4°C. Samples were centrifuged to separate the aqueous and organic phases, and the aqueous phase was subjected to a second phenol:chloroform:isoamyl alcohol extraction. DNA was then precipitated with isopropanol and sodium acetate at −20°C, washed with 100% ethanol, air-dried, and resuspended in 0.1× Tris-EDTA buffer. DNA concentration was measured using a NanoDrop.

Extracted DNA was submitted to SeqCenter for 16S rRNA sequencing. Libraries were prepared by amplifying the bacterial 16S rRNA gene V3–V4 region and were sequenced on an Illumina platform. Raw sequencing reads were processed and analyzed using QIIME 2 (Bolyen et al. 2019).

### Isolation and stimulation of bone marrow-derived neutrophils

Bone marrows were extracted from the femur and tibia of WT C57BL/6J mice, and neutrophils were isolated using the Neutrophil Isolation Kit with magnetic-activated cell sorting (MACS) (Miltenyi Biotec) following the manufacturer’s instructions. Neutrophil purity was validated by flow cytometry (>95%), and 10^5^ cells were seeded into a 96-well plate. The cells were then stimulated with LPS (100 μg/mL, Sigma-Aldrich), hBD2 (10 μM, Anaspec), hBD3 (10 μM, Anaspec) and PMA (100 μg/mL, Sigma-Aldrich) for 12 hours, and their supernatants were collected and stored at −80 °C.

### Enzyme-linked immunosorbent assay (ELISA)

To assay IL-17 release, the cell supernatants of stimulated neutrophils were assayed using the DuoSet ELISA Kit (R&D Systems) following the manufacturer’s protocol. The optical density was measured at 450 nm, with a wavelength correction at 540 nm, using a BioTek Synergy H1 microplate reader (Agilent). A standard curve was used to quantify the IL-17 released.

### LDH assay

Cell viability of stimulated bone marrow–derived neutrophils was assessed after 12 hours using the LDH Cytotoxicity Assay Kit II (ab65393) according to the manufacturer’s instructions. Absorbance was measured at 450 nm with wavelength correction at 650 nm using a BioTek Synergy H1 microplate reader (Agilent). Percent cell death was calculated according to the assay protocol.

### DRG isolation and culture

The spinal column of WT C57BL/6J, *Mrgpra3*^DTA^, and *Pirt*^Salsa^ mice aged 5-6 weeks was removed and placed in RPMI medium. DRGs were dissected and pooled in cold DH10 medium (DMEM/F12, 10%FBS, and 1% Penicillin-streptomycin). They were then digested in 1.1 mL of dispase/collagenase Type 1 mixture (dispase 4U/mL (Gibco), collagenase-1 342U/mL (Worthington CSL-1), prepared in 1X Hank’s balanced salt solution without phenol red)) and incubated for 30 minutes in a rotor. The neurons were then titrated around 20 times and filtered using a 70-micron filter. The neurons were then centrifuged at 500 rpm for 4 minutes and resuspended in DH10 medium. They were then plated on 8 × 8 mm coverslips coated with poly-D-lysine (0.1 mg/mL) and laminin (10 μg/mL) and incubated at 37 °C, and used within 20 hours for calcium imaging.

### *In vitro* calcium imaging

Cultured DRG neurons from *Pirt*^Salsa^ mice express a genetically encoded calcium indicator GCamp6f. DRGs from WT C57BL/6J and *Mrgpra3*^DTA^ were loaded with 5 μM Fluo-4 AM (Invitrogen) for 30 minutes in the dark. The neurons were then washed with calcium buffer (5M NaCl, 1M KCl, 0.5M CaCl_2_, 0.5M MgCl_2_, 10 mM HEPES, 20 mM Glucose, 1.19 mM NaHCO_3_, and 20.5 mM Sucrose, pH 7.4) and stimulated with hBD2 (10 μM), Chloroquine (1 mM), β-alanine (5 mM), Serotonin (5 μM), Capsaicin (30 μM) and KCl (100 mM). The fluorescence emission was detected at 488 nm, and the baseline fluorescence (F_0_) was defined as the average fluorescence intensity across the first 30 seconds of each imaging session. The maximum fluorescence (F_t_) was calculated as the average peak minus background fluorescence intensity within each ROI across the entire recording. ΔF/F was then computed as (F_t_ – F_0_)/F_0_. Neuronal activation was defined as a ratio of ΔF/F ≥ 50%. Each experiment contains 800-1000 cells and is analysed.

### DRG exposure surgery for *in vivo* calcium imaging

DRG exposure was performed as previously described (Zheng et al. 2022). Mice were anesthetized with intraperitoneal sodium pentobarbital (50-80mg/kg). After deep anaesthesia was reached, the animals’ back was shaved and aseptically prepared, and ophthalmic ointment (Lacrilube; Allergen Pharmaceuticals) was applied to the eyes to prevent drying. During the surgery, mice were kept on a heating pad (DC temperature controller, FHC) to maintain the body temperature at 37°C ± 0.5°C as monitored by a rectal probe.

### *In vivo* calcium imaging

In vivo imaging of whole L4 DRG was performed for 2 hours immediately following surgery. Images were acquired using a laser-scanning confocal microscope (Leica LSI microscope) equipped with a 5× 0.5 NA macro dry objective and fast EM-CCD camera. Live images were acquired at 10 frames in frame-scan mode per 6-7s, at depths of 0 to 70 mm below the dura with the DRG in the focal plane. Throughout imaging, body temperature was maintained at 37°C ± 0.5°C with a heating pad and rectal temperature monitoring. Anaesthesia was maintained with 2% isoflurane and pure oxygen delivered through nosecone. Mice were laid abdomen-down on a custom-designed microscope stage. The spinal column was secured at two sites using clamps. Imaging was monitored during the activation of DRG neuron cell bodies by peripheral stimuli (10μL of the stated concentration of either CQ or Human beta-defensin 2(HBD2) delivered via Hamilton syringe.

### *In vivo* calcium imaging data analysis

Raw image stacks were collected, deconvolved, and imported into ImageJ (NIH). Optical planes across sequential time points were realigned and motion-corrected using the StackReg rigid-body algorithm. Activated neuron counts across mice and imaging sessions were normalized to the total number of DRG neurons. The total number was counted using the thresholding function in ImageJ software from a high-quality, Z-stack image of DRG at baseline. The size of activated neurons was calculated using the area function in ImageJ.

Calcium signal amplitudes were expressed as (F_t_ – F_0_) /F_0_ as a function of time, i.e. ratio of fluorescence difference (F_t_ – F_0_) to basal value (F_0_). The average fluorescence intensity in the baseline period was taken as F_0_ and measured as the average pixel intensity during the first two to five frames of each imaging experiment. The maximum fluorescence intensity F_t_ was measured by calculating the average (peak – background) pixel values in a given region of interest (ROI) for each image frame recorded during the whole recording period. The F_t_ was then used to calculate ΔF/F using the formula ΔF/F = (F_t_ – F_0_) /F_0_. Activation of a neuron was defined as an increase in fluorescence intensity, ΔF/F ≥ 50%

### Statistical analysis

All statistical analyses were performed using Prism 10 (GraphPad). One-way and two-way ANOVA were used for multiple comparisons as indicated in the figure legends. Two-tailed unpaired Student’s t-test was used for data with a single comparison. All data are presented with mean ± standard deviation (SD). Statistical significance was assumed at p<0.05.

## Supplementary Material

1

2

3

4

5

6

7

8

9

10

11

## Figures and Tables

**Figure 1.**
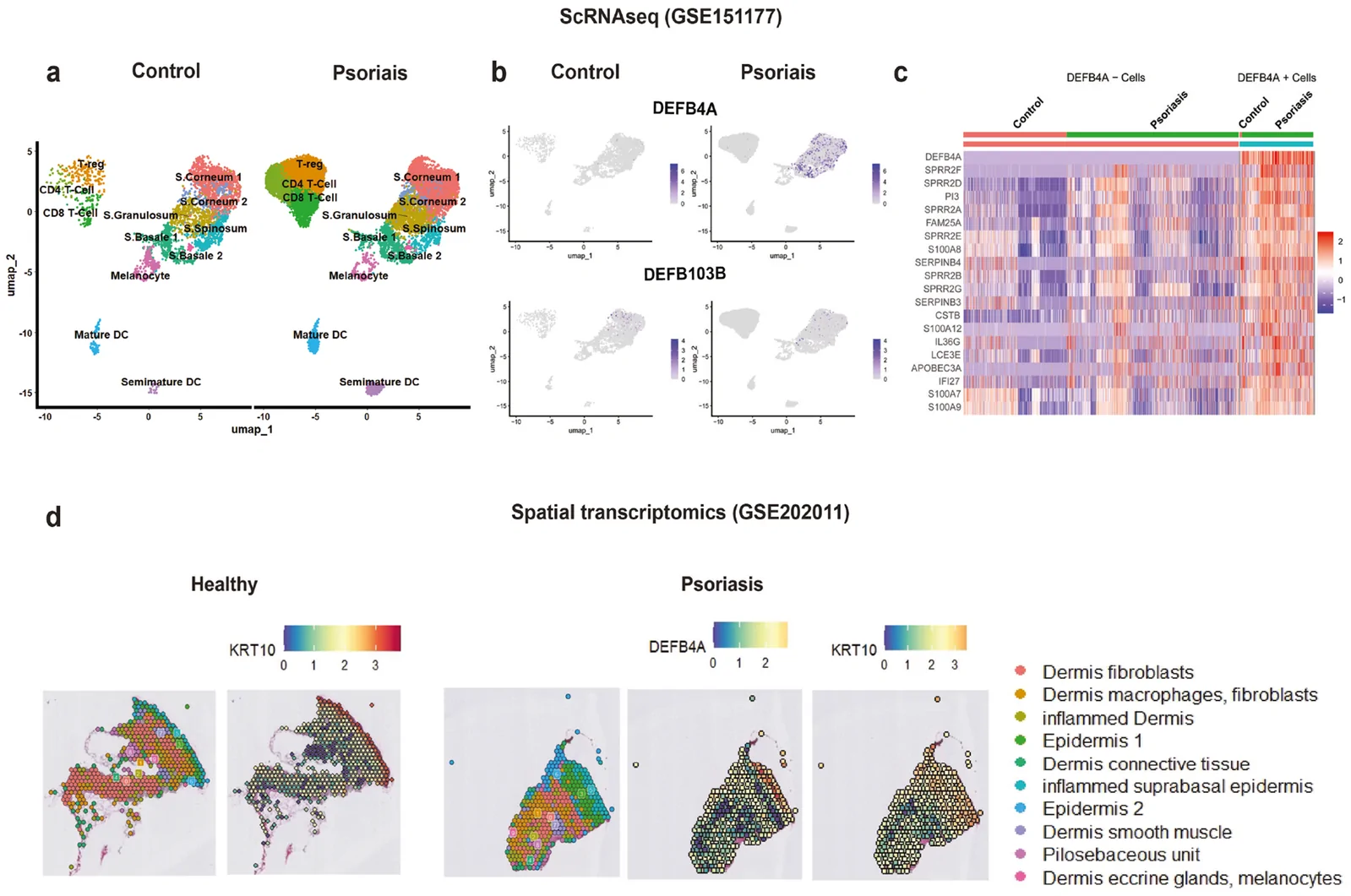
Defensins are highly expressed by inflamed suprabasal keratinocytes in human psoriatic skin. (**a**) UMAP analysis of published scRNA-seq data (GSE151177) showing major immune and keratinocyte subclusters from control and psoriatic skin. (**b**) Feature plots of *DEFB4A* and *DEFB103B*, demonstrating increased expression in keratinocyte subclusters in psoriatic skin. (**c**) Sub-clustering of keratinocytes into *DEFB4A−* and *DEFB4A*+ populations. The heatmap shows that *DEFB4A*+ cells co-express *S100*, *SPRR*, and *IL-36*. (**d**) Spatial transcriptomics (GSE202011), identifying epidermal and dermal subclusters. *DEFB4A* expression localizes to inflamed suprabasal keratinocytes in psoriatic, but not healthy, skin. *KERATIN 10* (*KRT10*) marks the suprabasal layer. scRNA-seq, single-cell RNA sequencing; UMAP, Uniform Manifold Approximation and Projection.

**Figure 2.**
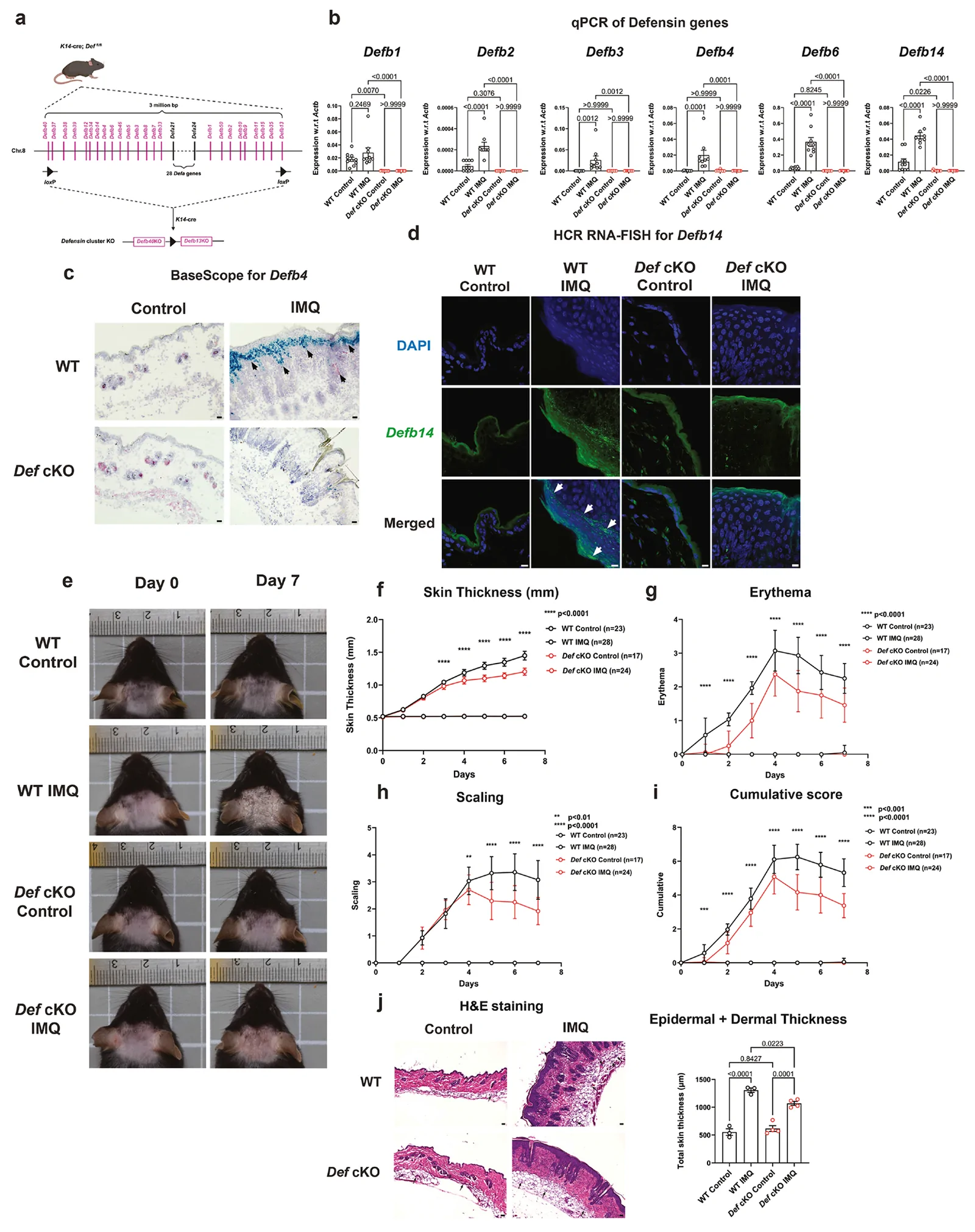
Defensins are required for psoriasis-like inflammation. (**a**) Schematic of keratinocyte-specific defensin cluster knockout (*Def* cKO) mice generated by inserting loxP sites flanking the β-defensin locus and crossing with *K14*-cre. (**b**) qPCR of β-defensin genes in WT (black) and *Def* cKO (red) control skin, and 7 days post IMQ treatment. (**c**) BaseScope showing *Defb4* (blue) in suprabasal epidermis of IMQ-treated WT skin and *Krt16* (red) in dermis. Scale bar, 50μm. (**d**) HCR RNA-FISH of *Defb*14 (green puncta) in IMQ-treated WT skin, not in *Def* cKO skin. Nuclei labelled with DAPI. Scale bar, 10μm. (**e**) Representative images of the shaved nape skin of control and IMQ-induced skin. (**f-i**) Quantification of skin thickness, erythema, scaling, and cumulative scores over 7 days. (**j**) H&E staining and quantification of total skin thickness (n=3). Scale bar, 50μm. Data is presented as mean ± SD. **p < 0.01, ***p < 0.001, ****p < 0.0001. One-way and two-way ANOVA were used to calculate the *p*-values. Sidak’s and Tukey’s multiple-comparison post hoc tests were used. WT, wildtype; IMQ, Imiquimod; HCR RNA-FISH, Hybridization chain reaction RNA fluorescence in situ hybridization; H&E, hematoxylin and eosin.

**Figure 3.**
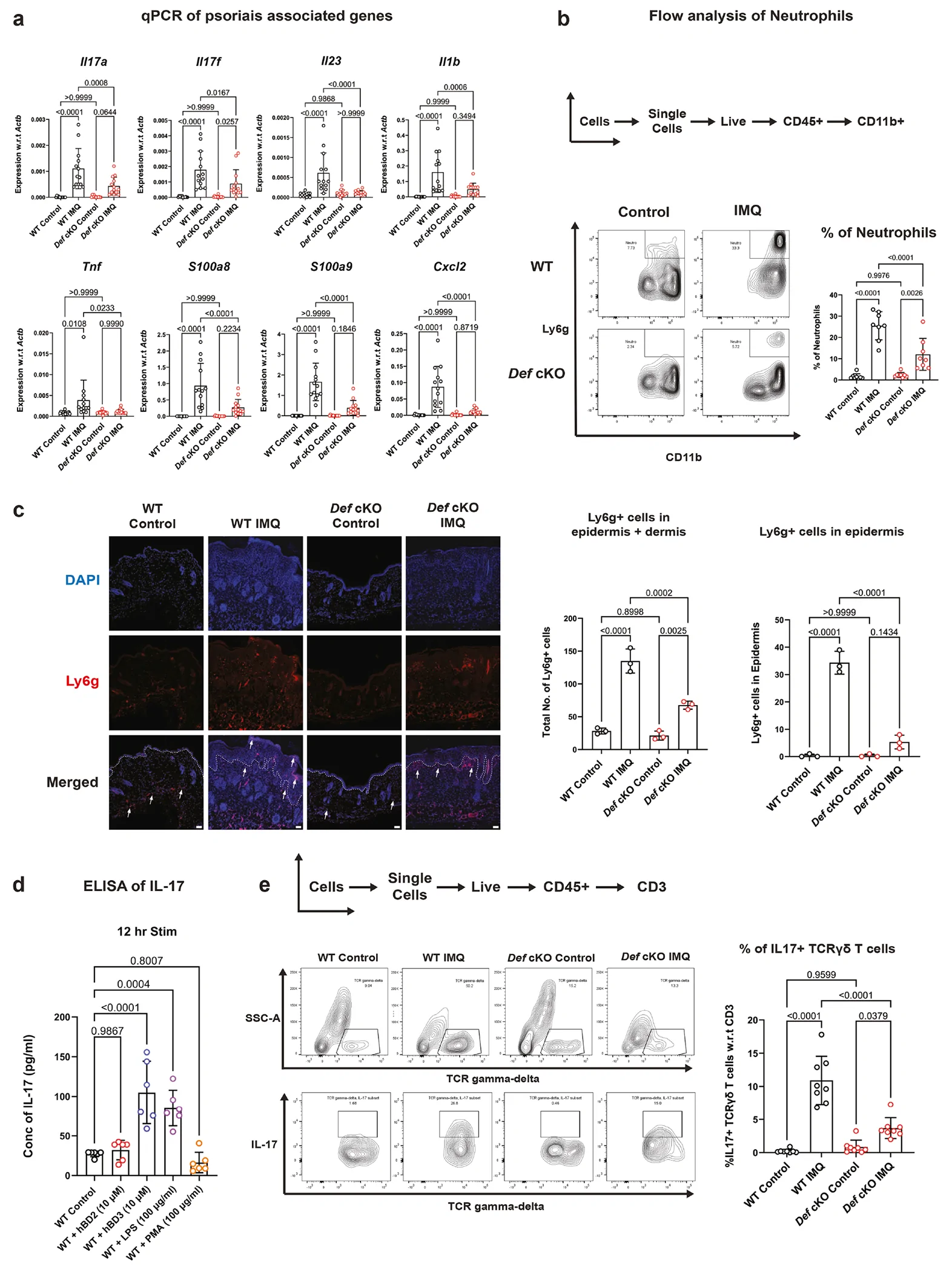
Defensins promote neutrophil recruitment and IL-17+ TCRγδ T cells infiltration in psoriasis. (**a**) qPCR analysis of proinflammatory (*Il17a*, *Il17f*, *Il23*, and *Tnf*) and neutrophil-associated genes (*Il1b*, *S100a8*, *S100a9,* and *Cxcl2)* in WT and *Def* cKO control and after 7 days of IMQ treatment. (n=13). (**b**) Flow cytometry analysis of neutrophils from WT control, WT IMQ, *Def* cKO control, and *Def* cKO IMQ-treated skin. The gating strategy of Live CD45^+^CD11^+^Ly6g^+^ neutrophils is shown. The quantification of neutrophil percentage is shown on the right (n=8). (**c**) Immunofluorescence images of Ly6g^+^ neutrophils (red) in WT control, WT IMQ, *Def* cKO control, and *Def* cKO IMQ-treated skin. Nuclei are labelled with DAPI. The quantification of total Ly6G^+^ cells in the skin and Ly6G^+^ cells within the epidermis is shown on the right. The dotted line indicates the epidermis, with arrows pointing to the infiltrated neutrophils (n=3). Scale bar, 50μm. (**d**) ELISA for IL-17 from the supernatant of bone marrow-derived neutrophils stimulated with LPS (100 μg/ml), hBD2 (10 μM) hBD3 (10 μM) and (100 μg/ml) PMA for 12 hours. (n=6). (**e**) Flow cytometry analysis of IL-17+ TCRγδ T cells in WT and *Def* cKO IMQ-treated skin. The quantification of total IL-17+ TCRγδ T cell percentage with respect to CD3 is shown on the right. (n=8). Data are presented as mean ± SD. One-way ANOVA was used to calculate the *p*-values. ELISA, enzyme-linked immunosorbent assay; LPS, lipopolysaccharide; hBD3, human β-defensin 3.

**Figure 4.**
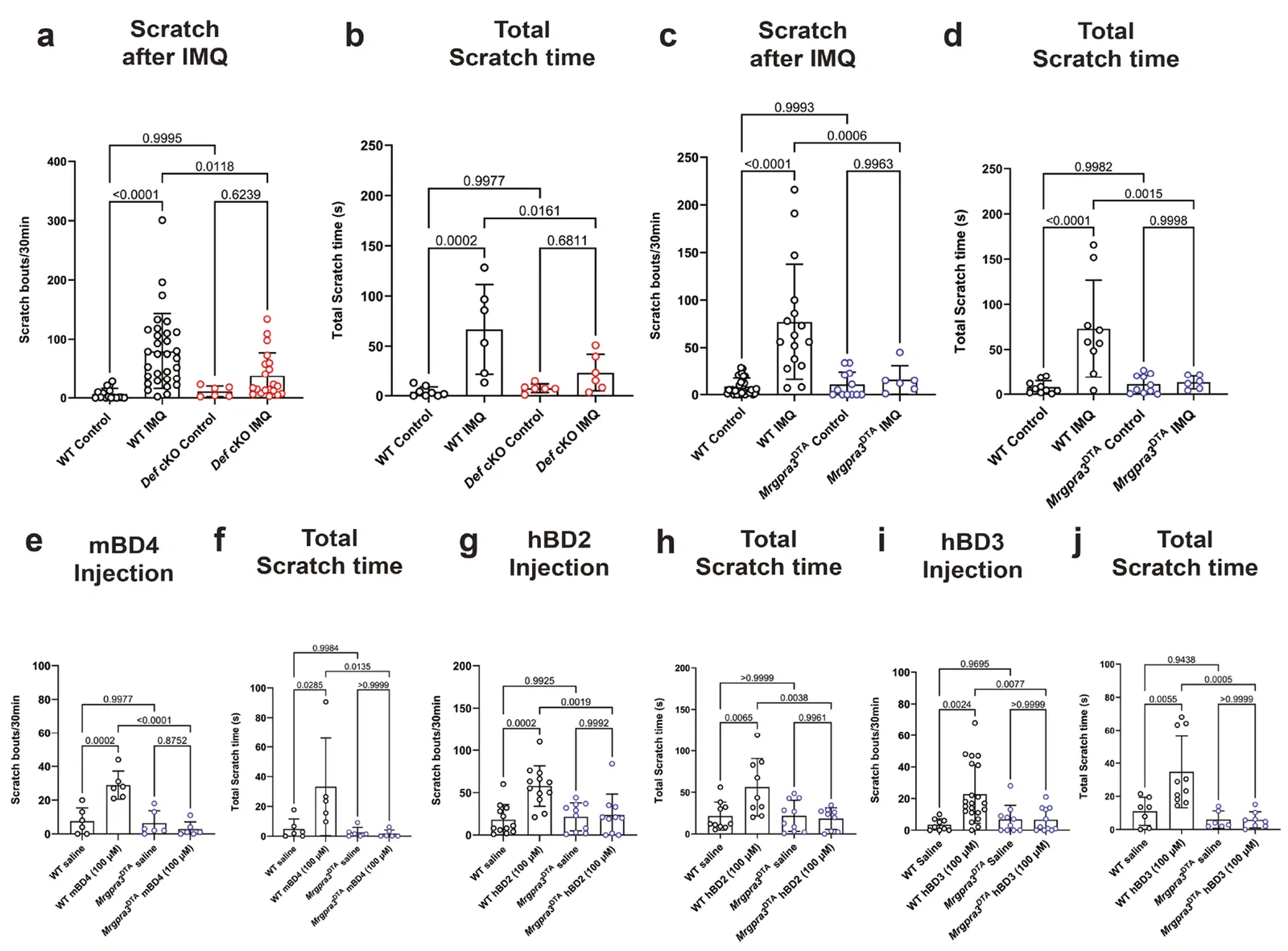
Defensins activate Mrgpra3 neurons to mediate psoriatic itch. (**a**) Quantification of scratching behaviour in WT (black) and *Def* cKO (red) mice treated with IMQ for 7 days. Scratch bouts were counted manually for 30 minutes. (n=6-31). (**b**) Total scratch time analysis of WT and *Def* cKO mice after 7 days of IMQ treatment using Scratch AID. The time was calculated using 30 frames per second. (n=6-8). (**c**) Quantification of itch behaviour in WT (black) and *Mrgpra3* neuron-ablated mice (*Mrgpra3*^DTA^) (blue) following 7 days of IMQ treatment. Scratch bouts were counted manually for 30 minutes. (n=6-31). (**d**) Total scratch time analysis of WT and *Mrgpra3*^DTA^ mice after 7 days of IMQ treatment using Scratch AID. (n=6-10). (**e**) Cheek injection of mBD4 (100 μM) in WT and *Mrgpra3*^DTA^ mice, and scratch bouts were counted for 30 minutes. (n=6). (**f**) Total scratch time analysis of WT and *Mrgpra3*^DTA^ mice after mBD4 (100 μM) cheek injection using Scratch AID. (n=5-6). (**g**) Cheek injection of hBD2 (100 μM) in WT and *Mrgpra3*^DTA^ mice, and scratch bouts were counted for 30 minutes. (n=10-12). (**h**) Total scratch time analysis of WT and *Mrgpra3*^DTA^ mice after hBD2 (100 μM) cheek injection using Scratch AID. (n=9-10). (**i)** Cheek injection of hBD3 (100 μM) in WT and *Mrgpra3*^DTA^ mice (n=9-21). (**j**) Total scratch time analysis of WT and *Mrgpra3*^DTA^ mice after hBD3 (100 μM) cheek injection using Scratch AID. (n=6-10). Data are presented as mean ± SD. One-way ANOVA and Sidak’s multiple comparison test were used to calculate the *p*-values. mBD4, mouse β-defensin 4; hBD2, human β-defensin 2; hBD3, human β-defensin 3; Mrgpr, Mas-related G-protein coupled receptor.

**Figure 5.**
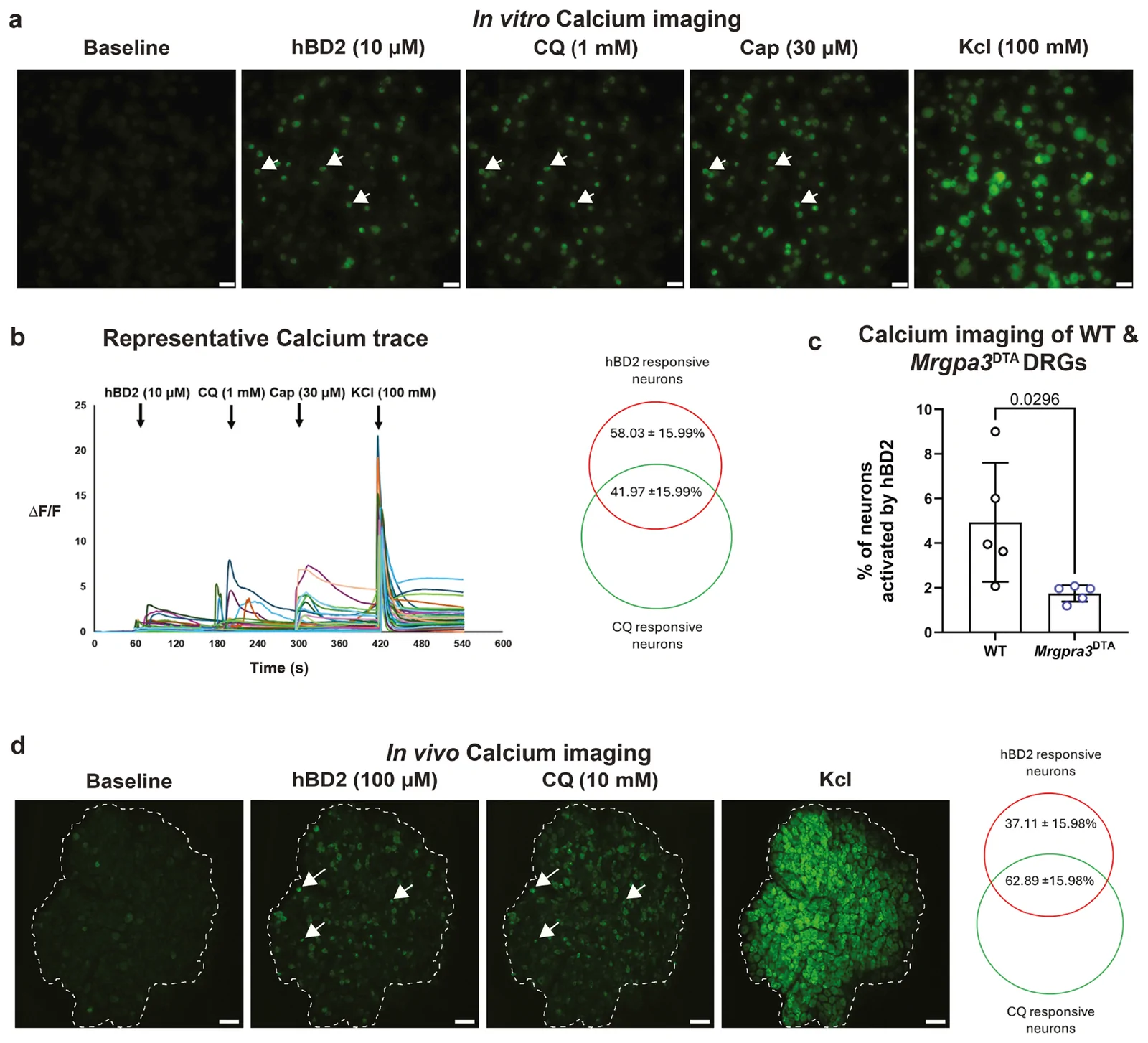
Human β-defensin 2 directly activates Mrgpra3+ sensory neurons, both in vitro and in vivo. (**a**) *In vitro* calcium images of cultured DRG neurons from *Pirt*^Salsa^ mice, which have a Gcamp6f (calcium indicator) genetically encoded. These neurons are stimulated with hBD2 (10 μM), chloroquine (CQ) (1 mM), capsaicin (cap) (30 μM), and KCl (100 mM). Scale bar, 50μm. (n=5). (**b**) Representative calcium trace of hBD2, CQ, cap-activated neurons. Arrows pointing at the neurons activated by hBD2, CQ, and cap. ΔF/F cutoff for activation is 0.5. Venn diagram showing that among hBD2-activated neurons, 41.97 ± 15.99% responded to CQ. (**c**) *In vitro* calcium imaging of DRG neurons from WT and *Mrgpra3*^DTA^ mice. The neurons are labelled with Fluo-4 for 30 minutes and stimulated with hBD2 (10 μM), chloroquine (CQ) (1 mM), capsaicin (cap) (30 μM), and KCl (100 mM). (n=5). (**d**) *In vivo* calcium images of L4 DRG from *Pirt*^Gcamp6s^ after hind paw injection of hBD2 and CQ. Arrows pointing at the neurons activated by both hBD2 and CQ. hBD2 evoked a strong response, and ~63% of hBD2-responsive neurons also respond to CQ. Scale bar, 50μm. (n=5). Data are presented as mean ± SD. Two-tailed *t*-test was usedto calculate the *p*-values.

## Data Availability

All data supporting the findings of this study are available from the corresponding authors upon request.
