# Supplementary material for: Defensins Mediate Skin Inflammation and Itch in Psoriasis
Source: J Invest Dermatol. Author manuscript; Available in PMC 2026 Oct 1. (PMC13625686; doi:10.1016/j.jid.2026.08.014)
Supplement: Supplementary file 1 [file NIHMS2210237-supplement-10.pdf]

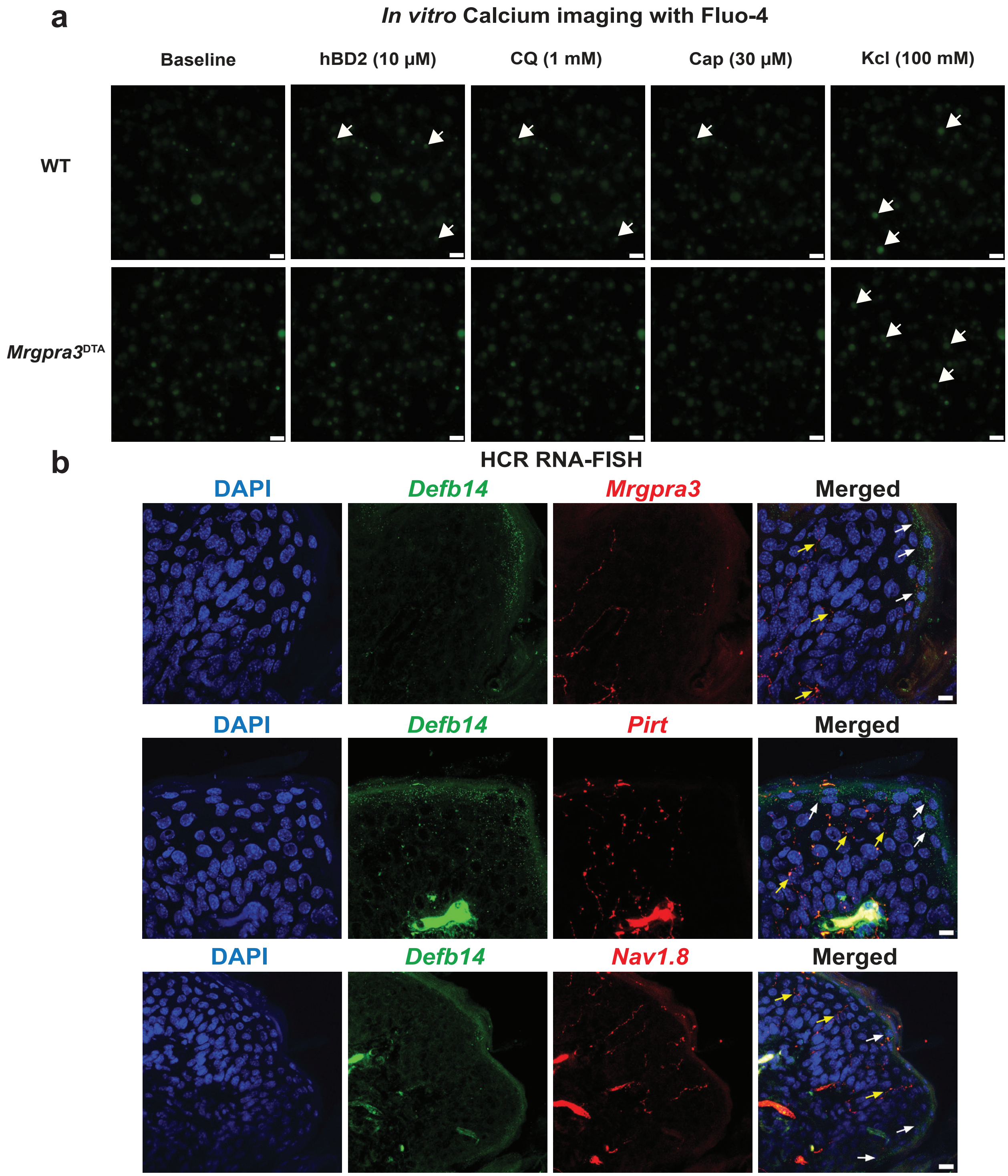

a
In vitro Calcium imaging with Fluo-4
Baseline
hBD2 (10 μM)
CQ (1 mM)
Cap (30 μM)
Kcl (100 mM)
WT
Mrgpra3DTA
b
HCR RNA-FISH
DAPI
Defb14
Mrgpra3
Merged
DAPI
Defb14
Pirt
Merged
DAPI
Defb14
Nav1.8
Merged
