# Supplementary material for: Defensins Mediate Skin Inflammation and Itch in Psoriasis
Source: J Invest Dermatol. Author manuscript; Available in PMC 2026 Oct 1. (PMC13625686; doi:10.1016/j.jid.2026.08.014)
Supplement: Supplementary file 2 [file NIHMS2210237-supplement-11.pdf]

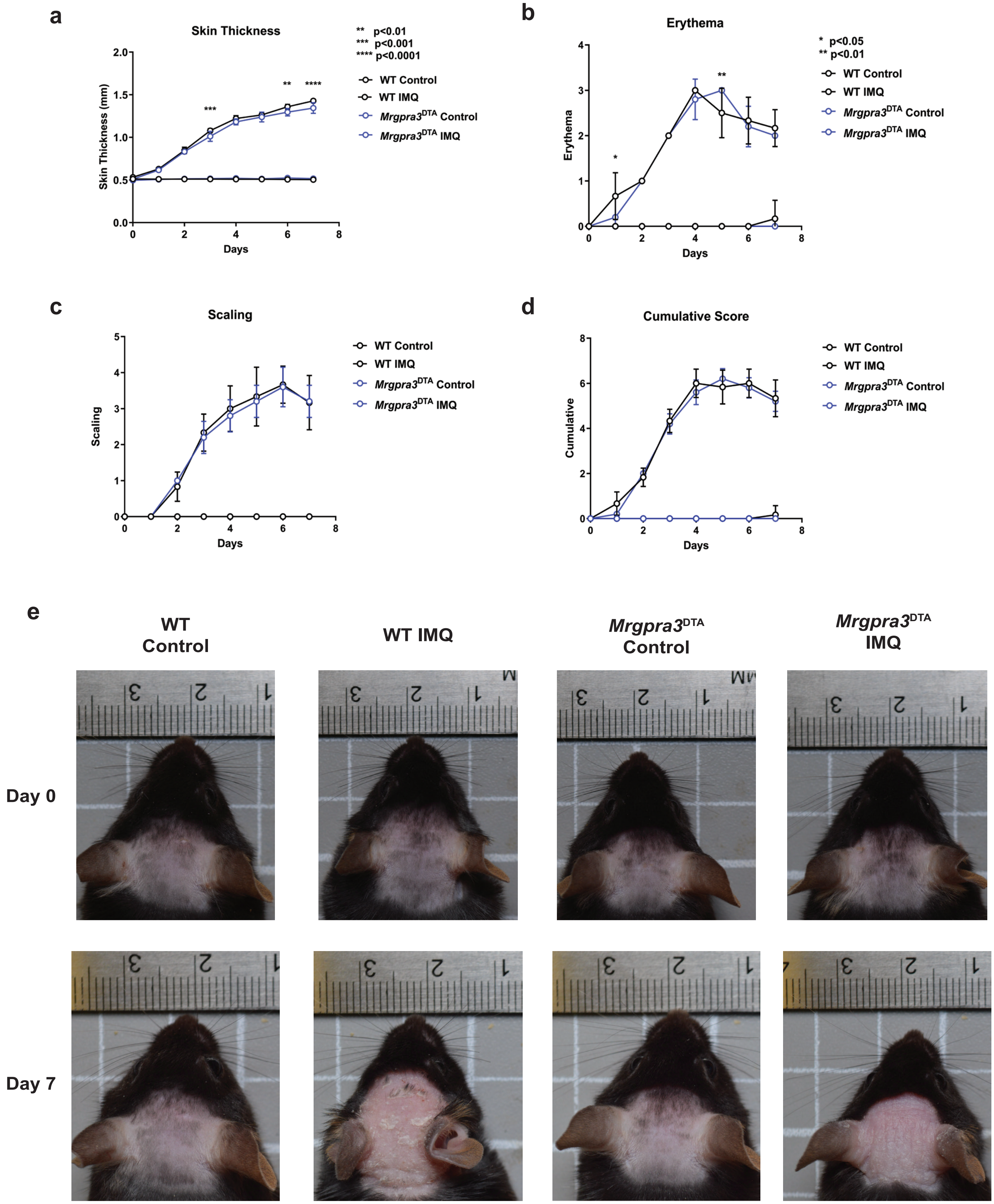
a
Skin Thickness
** p<0.01
*** p<0.001
**** p<0.0001
WT Control
WT IMQ
Mrgpra3DTA Control
Mrgpra3DTA IMQ
Skin Thickness (mm)
Days
b
Erythema
* p<0.05
** p<0.01
WT Control
WT IMQ
Mrgpra3DTA Control
Mrgpra3DTA IMQ
Erythema
Days
c
Scaling
WT Control
WT IMQ
Mrgpra3DTA Control
Mrgpra3DTA IMQ
Scaling
Days
d
Cumulative Score
WT Control
WT IMQ
Mrgpra3DTA Control
Mrgpra3DTA IMQ
Cumulative
Days
e
WT Control
WT IMQ
Mrgpra3DTA Control
Mrgpra3DTA IMQ
Day 0
Day 7
