# Supplementary material for: Defensins Mediate Skin Inflammation and Itch in Psoriasis
Source: J Invest Dermatol. Author manuscript; Available in PMC 2026 Oct 1. (PMC13625686; doi:10.1016/j.jid.2026.08.014)
Supplement: Supplementary file 3 [file NIHMS2210237-supplement-2.pdf]

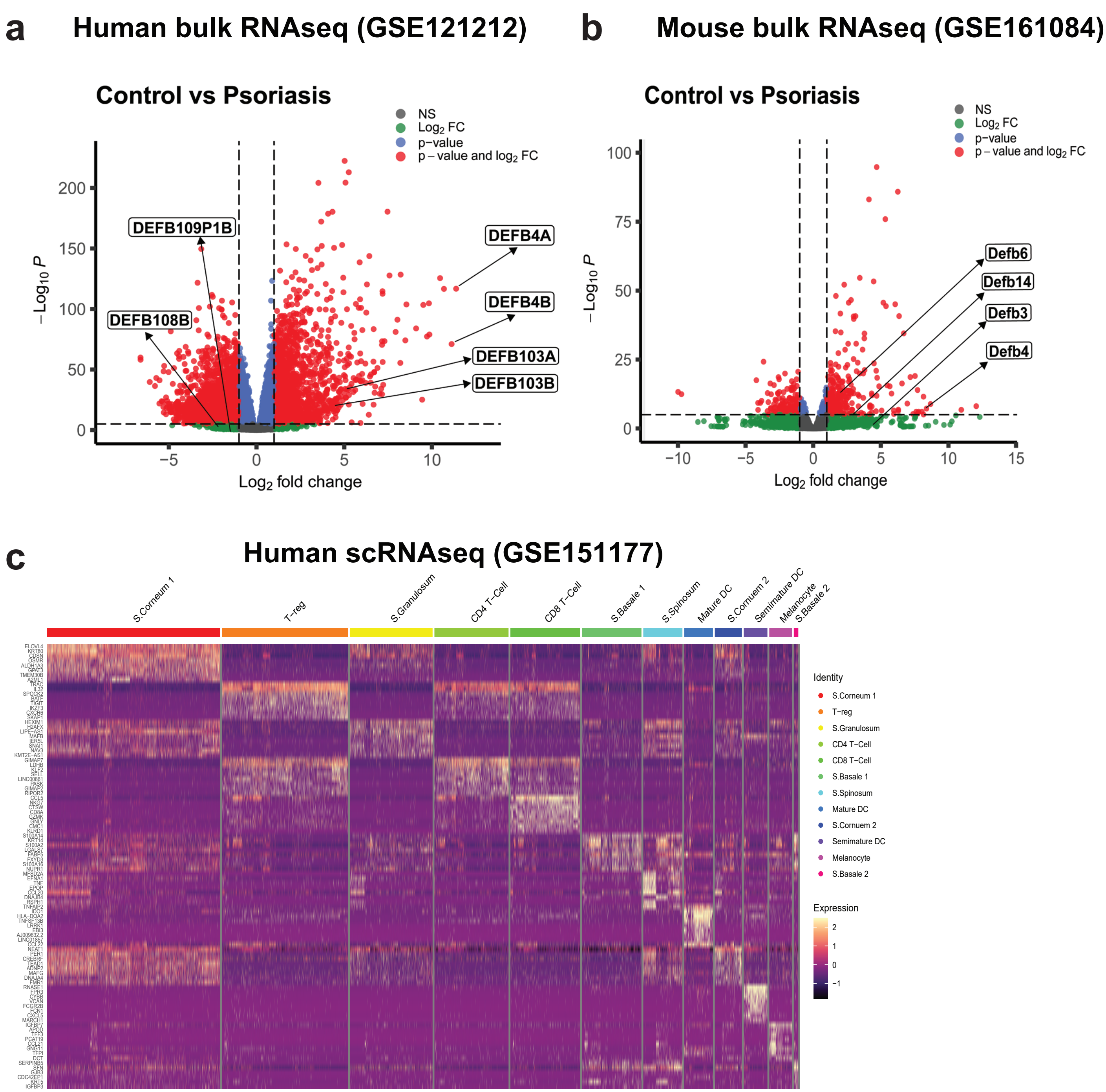

a
Human bulk RNAseq (GSE121212)
Control vs Psoriasis
NS
Log2 FC
p−value
p − value and log2 FC
DEFB109P1B
DEFB108B
DEFB4A
DEFB4B
DEFB103A
DEFB103B
− Log10 P
Log2 fold change
b
Mouse bulk RNAseq (GSE161084)
Control vs Psoriasis
Defb6
Defb14
Defb3
Defb4
c
Human scRNAseq (GSE151177)
S.Corneum 1
T-reg
S.Granulosum
CD4 T-Cell
CD8 T-Cell
S.Basale 1
S.Spinosum
Mature DC
S.Cornuem 2
Semimature DC
Melanocyte
S.Basale 2
Identity
Expression
