# Supplementary material for: Defensins Mediate Skin Inflammation and Itch in Psoriasis
Source: J Invest Dermatol. Author manuscript; Available in PMC 2026 Oct 1. (PMC13625686; doi:10.1016/j.jid.2026.08.014)
Supplement: Supplementary file 4 [file NIHMS2210237-supplement-3.pdf]

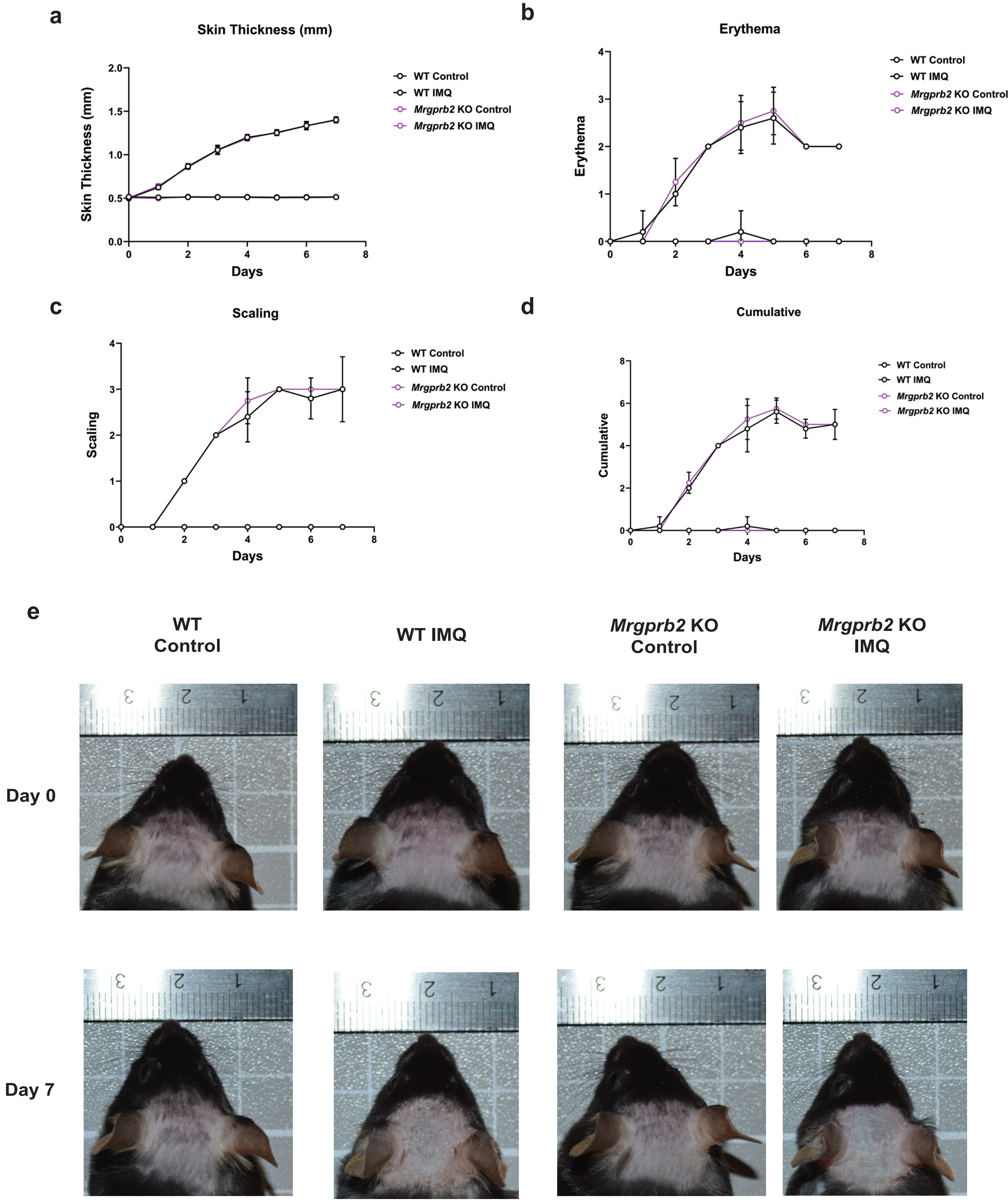
a
Skin Thickness (mm)
Skin Thickness (mm)
Days
WT Control
WT IMQ
Mrgprb2 KO Control
Mrgprb2 KO IMQ
b
Erythema
Erythema
Days
WT Control
WT IMQ
Mrgprb2 KO Control
Mrgprb2 KO IMQ
c
Scaling
Scaling
Days
WT Control
WT IMQ
Mrgprb2 KO Control
Mrgprb2 KO IMQ
d
Cumulative
Cumulative
Days
WT Control
WT IMQ
Mrgprb2 KO Control
Mrgprb2 KO IMQ
e
WT Control
WT IMQ
Mrgprb2 KO Control
Mrgprb2 KO IMQ
Day 0
Day 7
