# Supplementary material for: Defensins Mediate Skin Inflammation and Itch in Psoriasis
Source: J Invest Dermatol. Author manuscript; Available in PMC 2026 Oct 1. (PMC13625686; doi:10.1016/j.jid.2026.08.014)

a

Violin plot of DEFB4A and KRT10

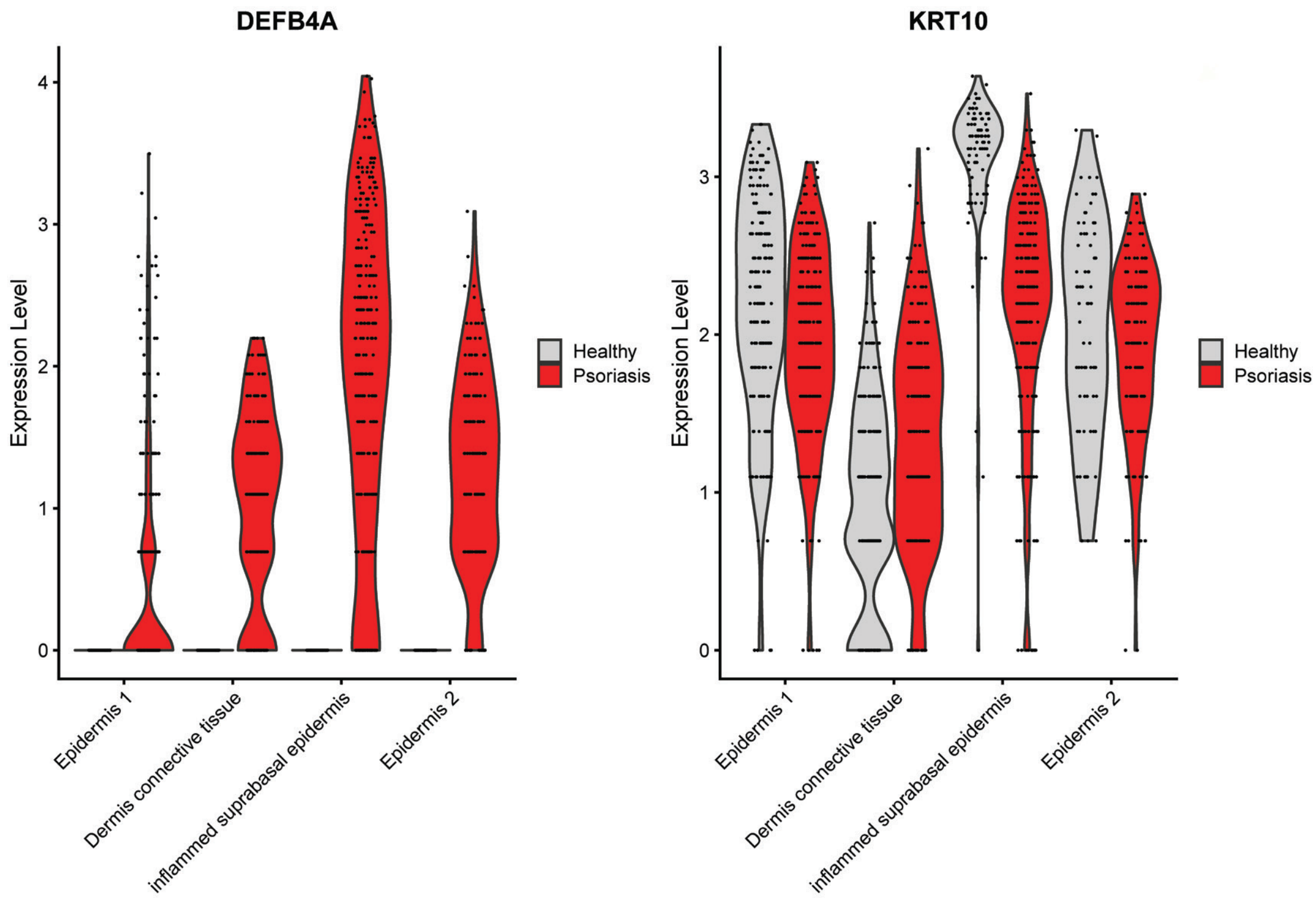

b

KRT10−DEFB4A spot−level correlation

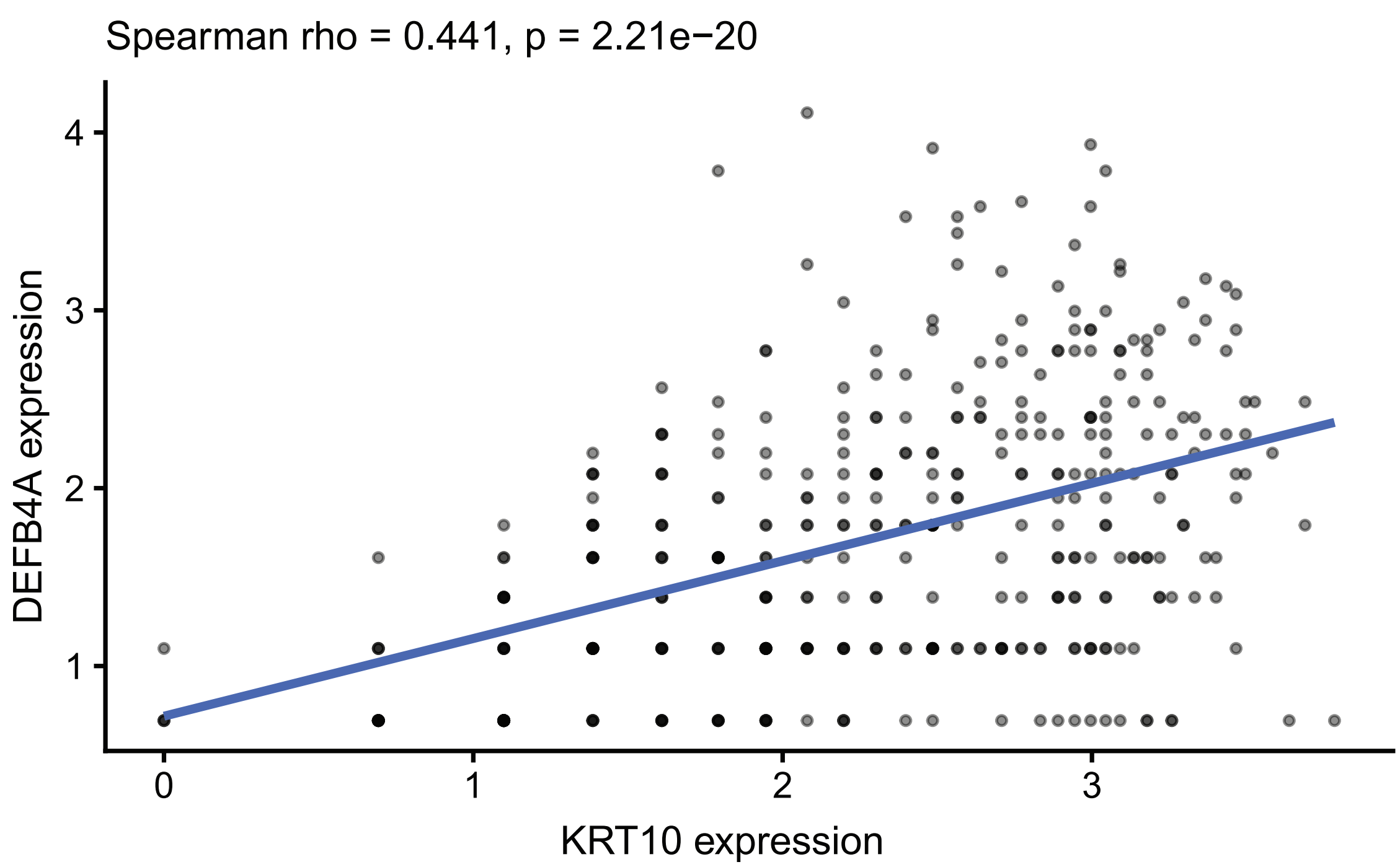

Supplement: Supplementary file 5 [file NIHMS2210237-supplement-4.pdf]
