# Supplementary material for: Defensins Mediate Skin Inflammation and Itch in Psoriasis
Source: J Invest Dermatol. Author manuscript; Available in PMC 2026 Oct 1. (PMC13625686; doi:10.1016/j.jid.2026.08.014)
Supplement: Supplementary file 6 [file NIHMS2210237-supplement-5.pdf]

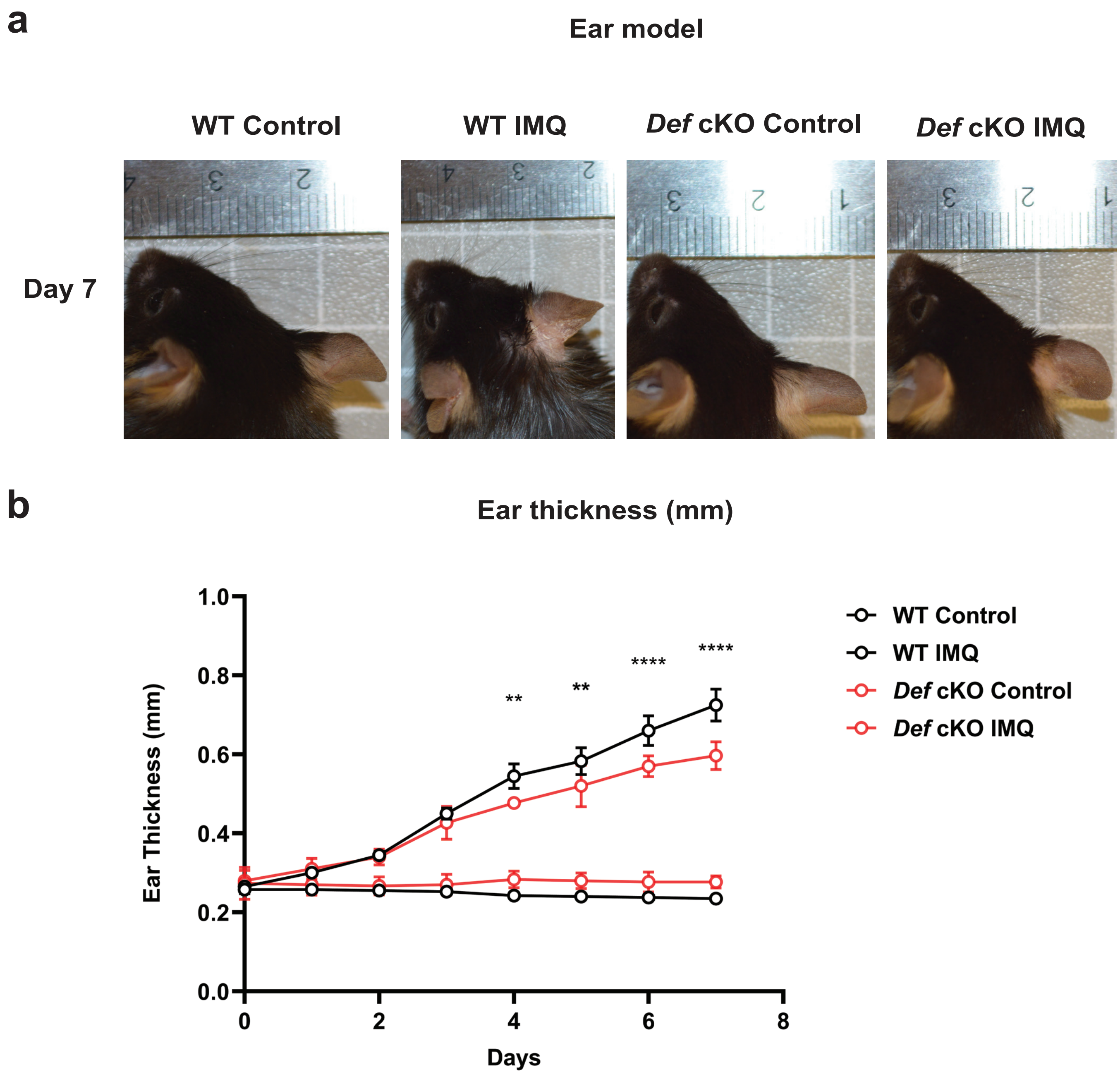
a
Ear model
WT Control
WT IMQ
Def cKO Control
Def cKO IMQ
Day 7
b
Ear thickness (mm)
Ear Thickness (mm)
1.0
0.8
0.6
0.4
0.2
0.0
0
2
4
6
8
Days
**
**
****
****
WT Control
WT IMQ
Def cKO Control
Def cKO IMQ
