# Supplementary material for: Defensins Mediate Skin Inflammation and Itch in Psoriasis
Source: J Invest Dermatol. Author manuscript; Available in PMC 2026 Oct 1. (PMC13625686; doi:10.1016/j.jid.2026.08.014)
Supplement: Supplementary file 7 [file NIHMS2210237-supplement-6.pdf]

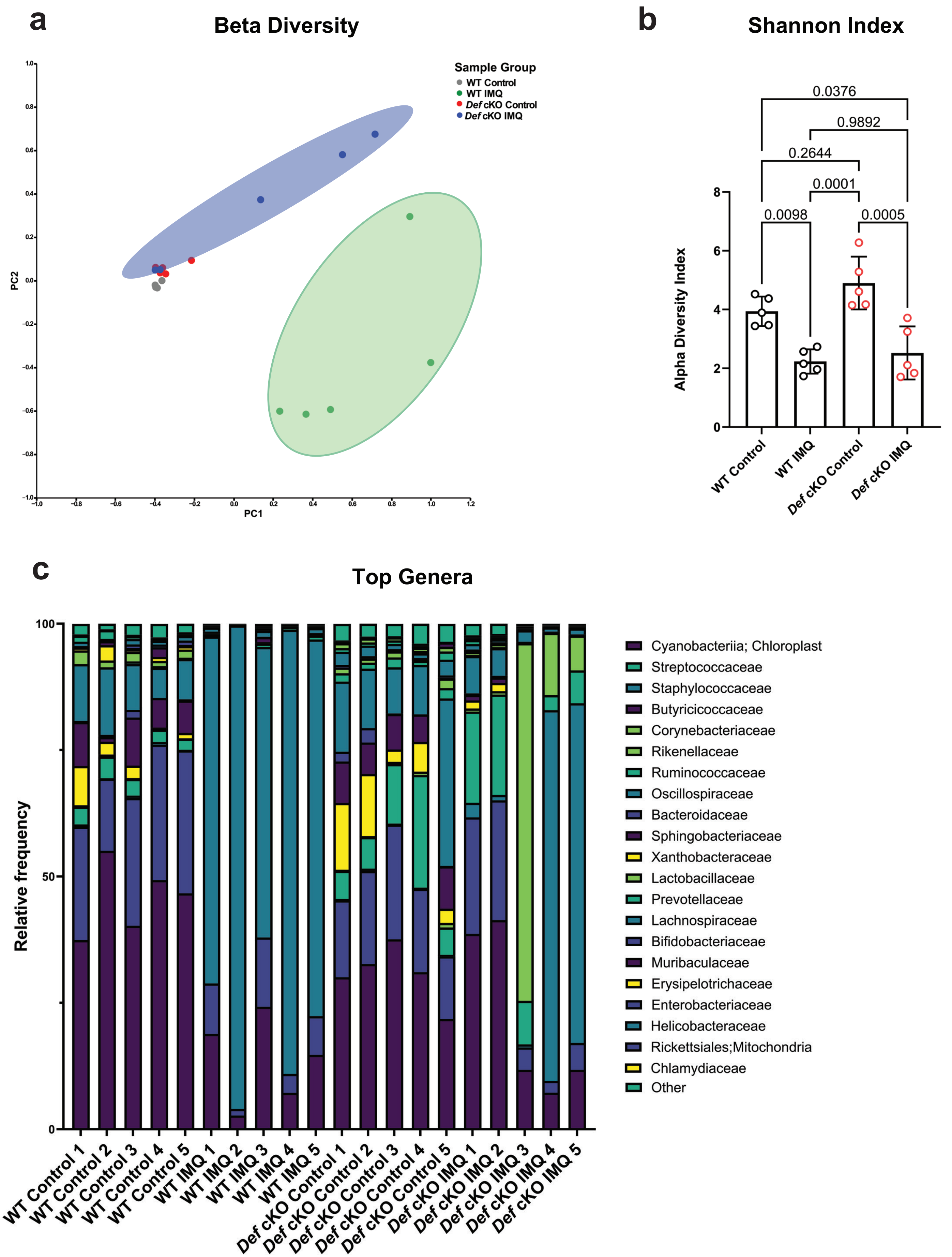
a
Beta Diversity
Sample Group
WT Control
WT IMQ
Def cKO Control
Def cKO IMQ
PC1
PC2
b
Shannon Index
Alpha Diversity Index
0.0376
0.9892
0.2644
0.0001
0.0098
0.0005
WT Control
WT IMQ
Def cKO Control
Def cKO IMQ
c
Top Genera
Relative frequency
Cyanobacteriia; Chloroplast
Streptococcaceae
Staphylococcaceae
Butyricicoccaceae
Corynebacteriaceae
Rikenellaceae
Ruminococcaceae
Oscillospiraceae
Bacteroidaceae
Sphingobacteriaceae
Xanthobacteraceae
Lactobacillaceae
Prevotellaceae
Lachnospiraceae
Bifidobacteriaceae
Muribaculaceae
Erysipelotrichaceae
Enterobacteriaceae
Helicobacteraceae
Rickettsiales;Mitochondria
Chlamydiaceae
Other
WT Control 1
WT Control 2
WT Control 3
WT Control 4
WT Control 5
WT IMQ 1
WT IMQ 2
WT IMQ 3
WT IMQ 4
WT IMQ 5
Def cKO Control 1
Def cKO Control 2
Def cKO Control 3
Def cKO Control 4
Def cKO Control 5
Def cKO IMQ 1
Def cKO IMQ 2
Def cKO IMQ 3
Def cKO IMQ 4
Def cKO IMQ 5
