# Supplementary material for: Defensins Mediate Skin Inflammation and Itch in Psoriasis
Source: J Invest Dermatol. Author manuscript; Available in PMC 2026 Oct 1. (PMC13625686; doi:10.1016/j.jid.2026.08.014)
Supplement: Supplementary file 8 [file NIHMS2210237-supplement-7.pdf]

LDH assay

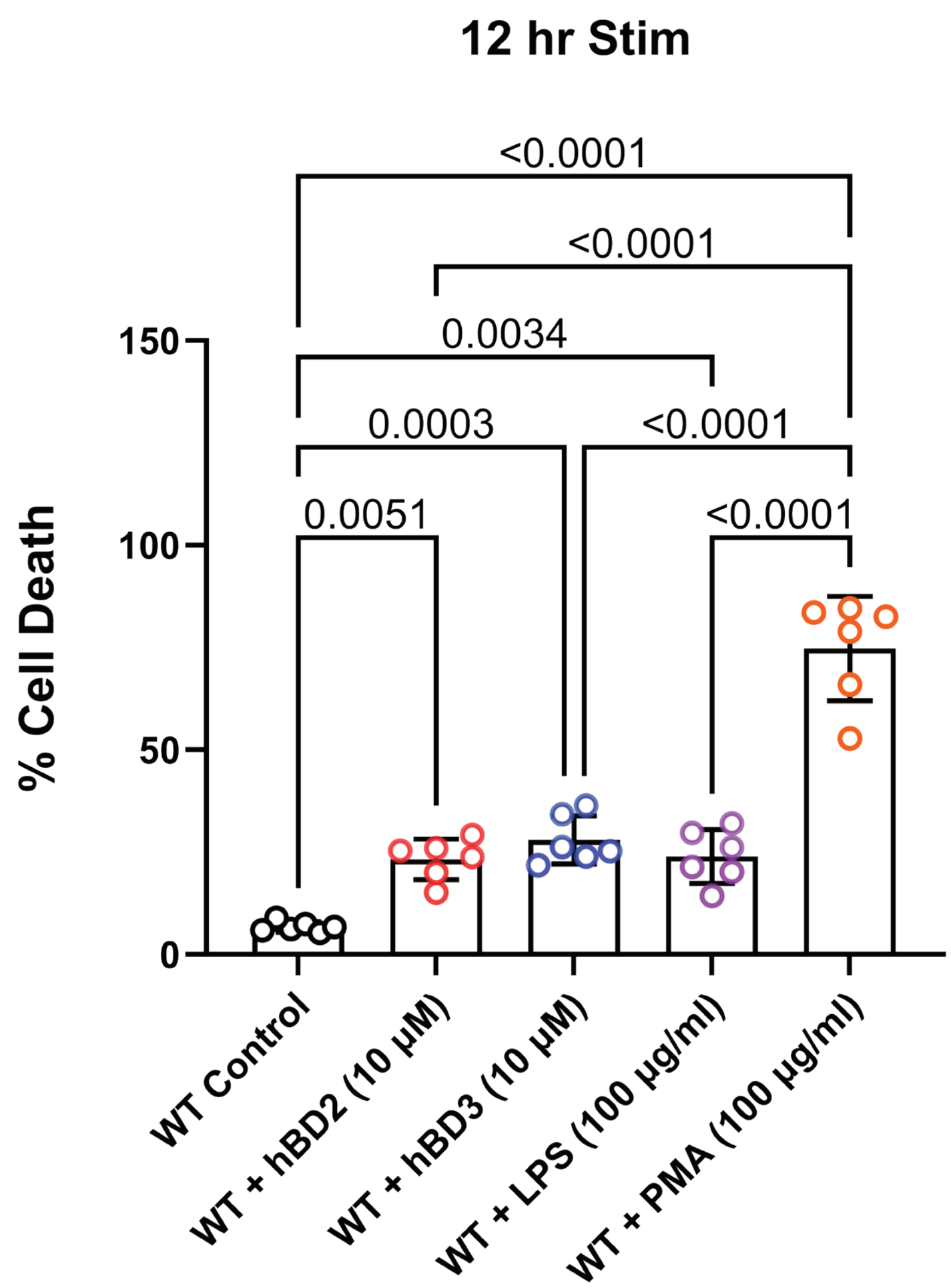

12 hr Stim
% Cell Death
150
100
50
0
<0.0001
<0.0001
0.0034
0.0003
<0.0001
0.0051
<0.0001
WT Control
WT + hBD2 (10 µM)
WT + hBD3 (10 µM)
WT + LPS (100 µg/ml)
WT + PMA (100 µg/ml)
