# Supplementary material for: Defensins Mediate Skin Inflammation and Itch in Psoriasis
Source: J Invest Dermatol. Author manuscript; Available in PMC 2026 Oct 1. (PMC13625686; doi:10.1016/j.jid.2026.08.014)
Supplement: Supplementary file 9 [file NIHMS2210237-supplement-8.pdf]

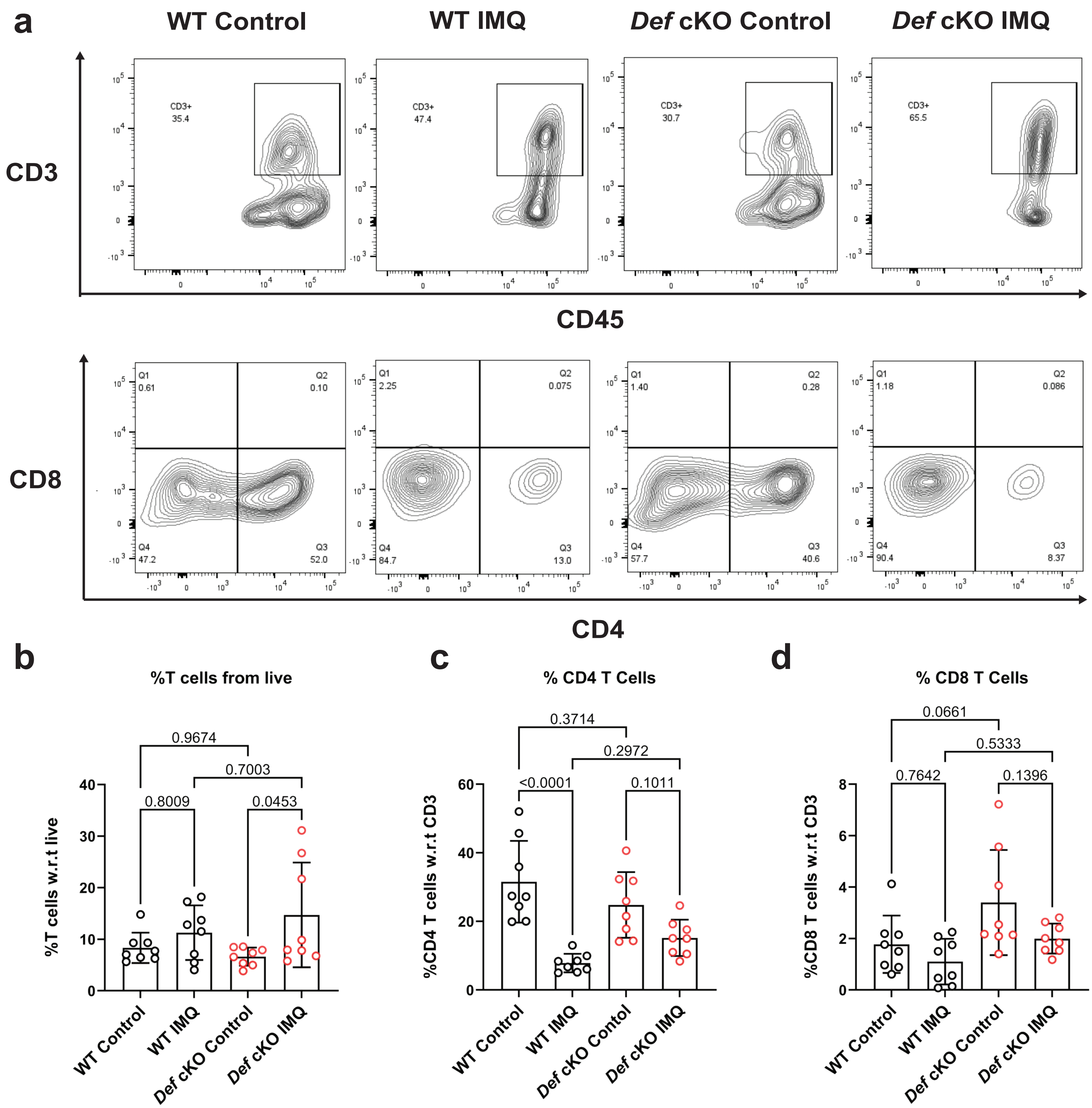

a
WT Control
WT IMQ
Def cKO Control
Def cKO IMQ
CD3
CD3+
35.4
CD3+
47.4
CD3+
30.7
CD3+
65.5
CD45
CD8
Q1
0.61
Q2
0.10
Q4
47.2
Q3
52.0
Q1
2.25
Q2
0.075
Q4
84.7
Q3
13.0
Q1
1.40
Q2
0.28
Q4
57.7
Q3
40.6
Q1
1.18
Q2
0.086
Q4
90.4
Q3
8.37
CD4
b
%T cells from live
%T cells w.r.t live
0.9674
0.7003
0.8009
0.0453
WT Control
WT IMQ
Def cKO Control
Def cKO IMQ
c
% CD4 T Cells
%CD4 T cells w.r.t CD3
0.3714
0.2972
<0.0001
0.1011
WT Control
WT IMQ
Def cKO Contol
Def cKO IMQ
d
% CD8 T Cells
%CD8 T cells w.r.t CD3
0.0661
0.5333
0.7642
0.1396
WT Control
WT IMQ
Def cKO Control
Def cKO IMQ
