# Supplementary material for: Defensins Mediate Skin Inflammation and Itch in Psoriasis
Source: J Invest Dermatol. Author manuscript; Available in PMC 2026 Oct 1. (PMC13625686; doi:10.1016/j.jid.2026.08.014)
Supplement: Supplementary file 10 [file NIHMS2210237-supplement-9.pdf]

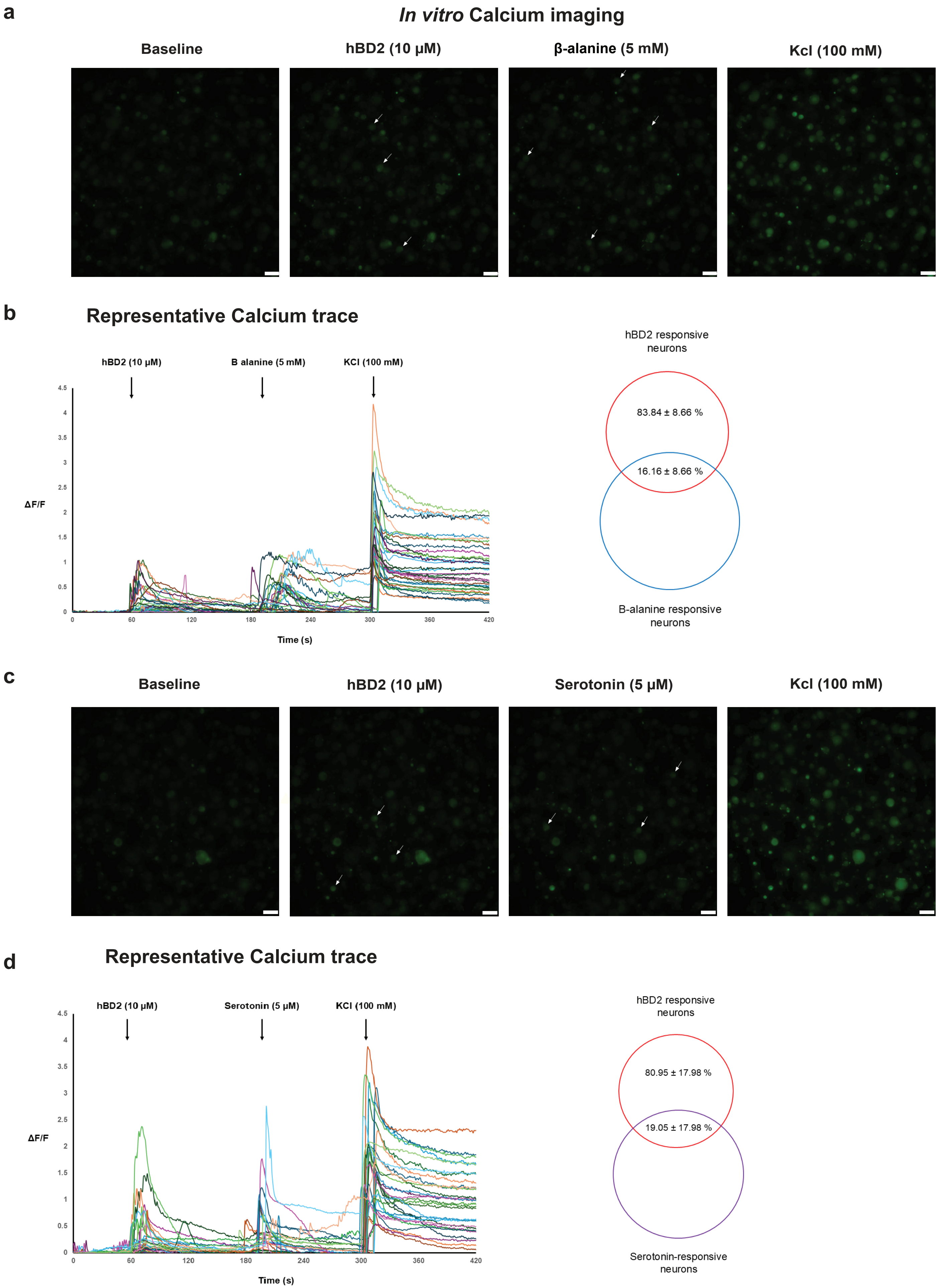

a
In vitro Calcium imaging
Baseline
hBD2 (10 µM)
β-alanine (5 mM)
Kcl (100 mM)
b
Representative Calcium trace
hBD2 (10 µM)
B alanine (5 mM)
KCl (100 mM)
ΔF/F
Time (s)
0
60
120
180
240
300
360
420
0
0.5
1
1.5
2
2.5
3
3.5
4
4.5
hBD2 responsive neurons
83.84 ± 8.66 %
16.16 ± 8.66 %
B-alanine responsive neurons
c
Baseline
hBD2 (10 µM)
Serotonin (5 µM)
Kcl (100 mM)
d
Representative Calcium trace
hBD2 (10 µM)
Serotonin (5 µM)
KCl (100 mM)
ΔF/F
Time (s)
hBD2 responsive neurons
80.95 ± 17.98 %
19.05 ± 17.98 %
Serotonin-responsive neurons
