# Supplementary material for: Defensins Mediate Skin Inflammation and Itch in Psoriasis
Source: J Invest Dermatol. Author manuscript; Available in PMC 2026 Oct 1. (PMC13625686; doi:10.1016/j.jid.2026.08.014)
Supplement: Supplementary file 11 [file NIHMS2210237-supplement-1.docx]

**SUPPLEMENTARY FIGURE LEGENDS**

**Supplementary Figure S1. Defensin expression is elevated in human and mouse psoriatic tissues.** (**a**) Volcano plot showing the differentially expressed genes (*DEFB4A* and *DEFB103B*) in human lesional psoriatic skin compared to control skin (GSE 121212). (**b**) Bulk RNA-seq analysis of IMQ-treated mouse skin (GSE161084). Volcano plot showing the differentially expressed genes (*Defb4*, *Defb3*, *Defb6*, and *Defb14*) in IMQ-treated skin compared to control skin. (**c**) Analysis of published human scRNA-seq data (GSE151177). Heatmap showing the top 8 differentially expressed genes in each subcluster; purple indicates low expression; orange indicates high expression.

**Supplementary Figure S2. Spatial transcriptomic analysis of *DEFB4A* and *KRT10* expression in psoriatic skin.** (**a**) Violin plots showing normalized *DEFB4A* and *KRT10* expression across annotated spatial clusters (Epidermis 1, Dermis connective tissue, Inflamed suprabasal epidermis and Epidermis 2) in healthy and psoriatic skin. *DEFB4A* expression is highest in the inflamed suprabasal epidermis, while *KRT10* is broadly expressed in all 4 clusters. (**b**) Spot-level correlation analysis between *DEFB4A* and *KRT10* expression across *DEFB4A*+ subclustered spatial transcriptomic spots. Spearman’s rho = 0.441, p = 2.21 × 10⁻^20^.

**Supplementary Figure S3. IMQ-treated *Def* cKO animals show reduced psoriatic inflammation in the ear model.** (**a**) Representative images of ears from control and IMQ-treated WT and *Def* cKO mice on day 7 of the ear psoriasis-like inflammation model. (**b**) Quantification of ear thickness over 7 days of IMQ treatment. (n=3-4). Data are presented as mean ± SD. ^**^*p* < 0.01,  ^∗∗∗∗^*p* < 0.0001. Two-way ANOVA and Tukey’s multiple comparison test were used to calculate the *p*-values.

**Supplementary Figure S4. IMQ treatment alters the skin microbiome in WT and *Def* cKO mice.** (**a**) Beta diversity analysis of 16S rRNA sequencing data from nape skin swabs collected from control and IMQ-treated WT and *Def* cKO mice. Principal coordinate analysis shows that WT control (grey) and *Def* cKO (red) control samples cluster closely together, whereas IMQ-treated WT (green) and *Def* cKO (purple) form distinct clusters. Each point represents an individual mouse sample. (**b**) Shannon alpha diversity index of skin microbiome samples from the control and IMQ-treated WT and *Def* cKO mice. (n=5). (**c**) Stacked bar charts depicting the relative abundance of the top bacterial genera detected by 16S rRNA sequencing across control and IMQ-treated WT and *Def* cKO skin samples. Data are presented as mean ± SD. One-way ANOVA was used to calculate the *p*-values.

**Supplementary Figure S5. Defensin stimulation does not induce significant cell death in bone marrow-derived neutrophils.** Lactate dehydrogenase (LDH) cytotoxicity assay of WT bone marrow-derived neutrophils following 12 hours of stimulation with hBD2 (10 μM), hBD3 (10 μM), LPS (100 μg/mL), and phorbol 12-myristate 13-acetate (PMA) (100 μg/mL). Percent cell death was quantified from LDH release in the culture supernatant. Data are presented as mean ± SD. One-way ANOVA was used to calculate the *p*-values.

**Supplementary Figure S6. Defensin does not affect the overall T cell populations in IMQ-induced psoriasis.** (**a**) Flow cytometric analysis of T cell subsets in WT and *Def* cKO skin after 7 days of IMQ treatment. The gating strategy is shown for CD45^+^CD3^+^ total T cells, followed by CD4^+^ and CD8^+^ T cells. (**b**) Quantification of total CD3^+^ T cells with respect to live in WT and *Def* cKO IMQ-treated skin (n=8). (**c-d**) Quantification of CD4^+^ T cells and CD8^+^ T cells with respect to CD3 in WT and *Def* cKO IMQ-treated skin (n=8). Data are presented as mean ± SD. One-way ANOVA was used to calculate the *p*-values

**Supplementary Figure S7. Defensin-responsive neurons show limited overlap with β-alanine- and serotonin-responsive neuronal populations.** (**a**) *In vitro* calcium images of cultured DRG neurons from WT mice. The neurons are labelled with Fluo-4 for 30 minutes and stimulated with hBD2 (10 μM), β-alanine (5 mM), and KCl (100 mM). Scale bar, 50μm. (n=3). (**b**) Representative calcium trace of hBD2, β-alanine, and KCl. Arrows pointing at the neurons activated by hBD2 and β-alanine. ΔF/F cutoff for activation is 0.5. Venn diagram showing that among hBD2-activated neurons, 16.16 ± 8.66% responded to β-alanine. (**c**) *In vitro* calcium images of cultured DRG neurons from WT mice. The neurons are labelled with Fluo-4 for 30 minutes and stimulated with hBD2 (10 μM), serotonin (5 μM), and KCl (100 mM). Scale bar, 50μm. (n=3). (**d**) Representative calcium trace of hBD2, serotonin, and KCl. Arrows pointing at the neurons activated by hBD2 and serotonin. ΔF/F cutoff for activation is 0.5. Venn diagram showing that among hBD2-activated neurons, 19.05 ± 17.98% responded to serotonin.

**Supplementary Figure S8. Defensins activate Mrgpra3 neurons and colocalize near the Mrgpra3 nerve terminals in psoriatic skin.** (**a**) Representative in vitro calcium images of cultured DRG neurons from WT and *Mrgpra3*^DTA^ mice loaded with calcium indicator Fluo-4. The neurons are stimulated with hBD2 (10 μM), chloroquine (CQ) (1 mM), capsaicin (cap) (30 μM), and KCl (100 mM). Arrows pointing at the neurons activated by hBD2, CQ, and cap. Scale bar, 50μm. (n=5). (**b**) Representative HCR RNA-FISH images of *Defb*14 transcripts (green puncta) in IMQ-treated *Mrgpra3*^Tdtomato^, *Pirt*^Tdtomato,^ and *Nav1.8*^Tdtomato^, in which the Mrgpra3, Pirt, and Nav1.8 neurons express Tdtomato. Neurons are shown in red and nuclei labelled with DAPI (n=3). Scale bar, 50μm.

**Supplementary Figure S9. Mrgpra3 neurons are not required for psoriatic inflammation.** (**a-d**) Quantification of IMQ-induced skin pathology in WT and *Mrgpra3*^DTA^ mice across 7 days of treatment. (**a**) Skin thickness (**b**), Erythema scores (**c**), Scaling scores (**d**), Cumulative scores from WT control (n=6), WT IMQ (n=6), *Mrgpra3*^DTA^ control (n=3), and *Mrgpra3*^DTA^ IMQ (n=5) mice across 7 days. (**e**) Representative images of shaved nape skin from WT and *Mrgpra3*^DTA^ control and IMQ-treated on days 0,3,5, and 7. Data is presented as mean ± SD. ^∗^*p* < 0.05, ^**^*p* < 0.01, ^∗∗∗^*p* < 0.001,^∗∗∗∗^*p* < 0.0001. Two-way ANOVA was used to calculate the *p*-values.

**Supplementary Figure S10. Mrgprb2 is not necessary for IMQ-induced psoriatic inflammation. (a-d)** Quantification of IMQ-induced skin pathology in WT and *Mrgprb2* KO mice across 7 days of treatment. (**a**) Skin thickness (**b**), Erythema scores (**c**), Scaling scores (**d**), Cumulative scores from WT control (n=5), WT IMQ (n=5), *Mrgprb2* KO control (n=4), and *Mrgprb2* KO IMQ (n=4) mice across 7 days. (**e**) Representative images of shaved nape skin from WT and *Mrgprb2* KO control and IMQ-treated on days 0, and 7. Data is presented as mean ± SD. Two-way ANOVA was used to calculate the *p*-values.
